# Boosting the OER performance of NiFe_2_O_4_ through Cr and Mn doping via hydrothermal synthesis

**DOI:** 10.3389/fchem.2026.1778233

**Published:** 2026-04-21

**Authors:** Davide Vendrame, Soufiane Boudjelida, Enrico Negro, Paolo Dolcet, Vito Di Noto, Silvia Gross

**Affiliations:** 1 Università degli Studi di Padova, Dipartimento di Scienze Chimiche, Padova, Italy; 2 Section of “Chemistry for the Technology” (ChemTech), Department of Industrial Engineering, University of Padova, Padova, Italy; 3 Centro Studi di Economia e Tecnica dell’Energia “Giorgio Levi Cases”, Padova, Italy; 4 Consorzio Interuniversitario Nazionale per la Scienza e Tecnologia dei Materiali (INSTM), Firenze, Italy; 5 Karlsruher Institut für Technologie, Institut fur Technische Chemie und Polymerchemie, Karlsruhe, Germany

**Keywords:** oxygen evolution reaction, electrocatalysis, water splitting, ferrites, spinel oxides, hydrothermal synthesis, chromium doping, manganese doping

## Abstract

The growing demand for green hydrogen requires efficient, cost-effective electrocatalysts for the oxygen evolution reaction (OER), a process currently hindered by sluggish kinetics. This study explores the optimisation of the spinel oxide NiFe_2_O_4_ through the partial Fe substitution with Cr and Mn, synthesised via a subcritical hydrothermal method, as an alternative to the standard Pt-group metals (PGM)-based electrocatalysts for the OER in alkaline environment. The work aims to establish a direct correlation between the chemical nature of the dopant, the resulting physicochemical properties, and the electrocatalytic performance. Detailed structural and surface characterisation, including XRD, TEM, and XPS, revealed distinct behaviours for the two dopants. Cr incorporation successfully produced phase-pure spinel nanoparticles with significantly reduced crystallite sizes and very high specific surface area (up to 226 m^2^/g). In contrast, high Mn substitution led to the formation of secondary phases (Ni(OH)_2_) and nanoscale inhomogeneity, which persisted even after calcination, suggesting an incomplete inclusion of the three different metals in the same spinel lattice. Electrochemical investigations demonstrated that the nature of the dopant strongly influences OER activity. While Mn-doped samples showed higher apparent activity than pristine NiFe_2_O_4_, this improvement was attributed solely to an increased number of active sites (surface area) rather than improved intrinsic kinetics. Conversely, the Cr-substituted sample NiFeCrO_4_ exhibited superior performance, surprisingly matching the OER performances of the benchmark IrO_X_. This outstanding activity was ascribed to a synergistic effect: the material combines a high specific surface area with enhanced intrinsic kinetics, driven by an optimal composition rich in Cr^3+^ which is hypothesised to modulate the overall $e_{g}$ occupation to a favourable value for promoting the OER.

## Graphical Abstract

**Figure:**
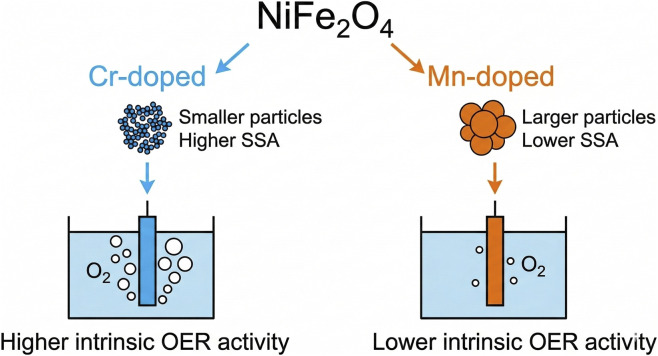

## 1 Introduction

The growing demand for sustainable energy has accelerated the development of water-splitting technologies for green hydrogen production (Atanassov et al., 2021; Di Noto, 2024). A significant bottleneck in this process is the kinetically sluggish oxygen evolution reaction (OER), which necessitates efficient, stable, and cost-effective electrocatalysts (Atanassov et al., 2021; Buttler and Spliethoff, 2018; Crabtree and Dresselhaus, 2008; Gao et al., 2024; Muhyuddin et al., 2025; Rego de Vasconcelos and Lavoie, 2019; Santoro et al., 2022; Zainal et al., 2024). Although Pt-group metal (PMG) oxides such as RuO_2_ and IrO_2_ are highly active, their scarcity and high cost hinder their large-scale application (Shi et al., 2019; Yu et al., 2019; Luo et al., 2020; Plevová et al., 2021; Li et al., 2020; Lee et al., 2012). Consequently, research has intensified on earth-abundant, first-row transition-metal-based materials as viable alternatives (Santoro et al., 2022; Gao et al., 2024; Zainal et al., 2024; Plevová et al., 2021; Xie et al., 2022; Roger et al., 2017; Miller et al., 2020; Araújo et al., 2024; McCrory et al., 2013). Among these, spinel oxides with the general formula AB_2_O_4_ have emerged as a highly promising class of OER electrocatalysts, owing to their compositional flexibility, inherent stability, and tunable electronic properties (Avcı et al., 2022; Olowoyo and Kriek, 2022; Landon et al., 2012; Li and Selloni, 2014; Si et al., 2017; Li et al., 2015; Simon et al., 2021; Chen et al., 2019; Alamro et al., 2024; Huang et al., 2018; Peng et al., 2025; Xie et al., 2019; Ahmed et al., 2025; Gong and Dai, 2015; Xu et al., 2023; Kim et al., 2018; Vedanarayanan et al., 2025; Fabbri et al., 2014; He et al., 2025a). The performance of spinel oxides as catalysts for the OER is intrinsically linked to their electronic structure, specifically the occupancy of the $e_{g}$ orbitals of the active transition-metal cations in the octahedral sites (O_h_) (Triolo et al., 2024; Triolo et al., 2025; Vezzù et al., 2025). As the $e_{g}$ orbital has a strong spatial overlap with oxygen-related OER intermediates, the binding strength of the metal–oxygen bond is highly influenced by the $e_{g}$ occupancy (Triolo et al., 2024). In contrast, tetrahedral (T_d_) sites are generally considered less active due to a lower orbital overlap with the oxygen-related OER intermediates (Suntivich et al., 2011; Wei et al., 2017; Zhou et al., 2019; Sun et al., 2020; Li et al., 2018). According to this catalytic descriptor, the OER activity reaches the maximum when the $e_{g}$ filling reaches unity ( $e_{g}^{1}$ ), indicating an optimal bond strength (Suntivich et al., 2011). Nickel ferrite (NiFe_2_O_4_), in particular, has attracted significant attention for its OER activity and stability in alkaline media (Gong and Dai, 2015; Gong et al., 2021; McCrory et al., 2013; Simon et al., 2021). However, according to the $e_{g}$ filling descriptor, the Ni^2+^ ( $t_{2g}^{6}e_{g}^{2}$ ) and Fe^3+^ ( $t_{2g}^{3}e_{g}^{2}$ ) cations in the O_h_ sites result in a non-ideal electronic configuration for OER. Therefore, tailoring the material electronic properties through doping is a powerful strategy to modulate the electronic structure in terms of $e_{g}$ orbital filling, thereby boosting the intrinsic activity (Wang et al., 2023; Fabbri et al., 2014; Hussain et al., 2024; Liu et al., 2021; Zhai et al., 2025; Han et al., 2016). The partial substitution of Fe^3+^ cations in the NiFe_2_O_4_ lattice presents a direct route to fine-tune this electronic configuration. In this context, Cr and Mn are considered particularly effective dopants. The introduction of Cr^3+^ ( $t_{2g}^{3}e_{g}^{0}$ ) is hypothesised to modulate the local coordination environment, thereby lowering the overall $e_{g}$ filling of the cations in the O_h_ sites, bringing it closer to the optimal value of unity for OER catalysis (Liu et al., 2024). For example, in ZnFe_2-X_Cr_X_O_4_, the presence of Cr^3+^ in O_h_ sites, along with Fe^3+^, tunes the $e_{g}$ occupancy of the transition metals in O_h_ enhancing the overall OER activity (Li et al., 2018). This effect is hypothesised to occur through the superexchange interaction between neighbouring O_h_-O_h_ sites (Wei et al., 2017; Wang et al., 2023; Khomskii and Sergey, 2021). Conversely, Mn is a well-known active component in OER catalysts, valued for its accessible multiple oxidation states (Mn^2+^, Mn^3+^, Mn^4+^), which can provide a flexible redox pathway for the OER mechanism (Wei et al., 2017; Ahmed et al., 2023; Yang et al., 2023; Zhu et al., 2013; Rios et al., 1998). Doping with Mn can introduce new active sites and facilitate charge transfer processes, although its effect on the overall $e_{g}$ filling is more complex due to its variable oxidation states and coordination site preferences within the spinel lattice.

While the concept of Cr and Mn doping in NiFe_2_O_4_ has been experimentally already explored to modulate its electronic structure for the OER (Gan et al., 2022; Shah et al., 2026), the present study introduces key novel findings regarding the synthetic methodology, the resulting nanostructuring, and the systematic decoupling of morphological properties from intrinsic catalytic activity in determining the overall OER performance.

In this study, we synthesised a series of NiFe_2-X_M_X_O_4_ (M = Cr, Mn) spinel oxides using a subcritical hydrothermal method at relatively low temperature, adapted from our previous works (Diodati et al., 2014; Dolcet et al., 2018). This wet-chemistry approach offers several advantages over traditional high-temperature solid-state synthesis, including lower energy consumption, the use of water as solvent and, crucially, the unique ability to obtain very small nanoparticles (down to <5 nm) with an exceptionally high specific surface area (up to 226 m^2^/g). The materials were synthesised with varying degrees of Fe substitution to systematically investigate the impact of doping on the structural and electrochemical properties. This study aims to establish a direct correlation between the dopant chemical nature (Cr vs. Mn), the resulting physicochemical properties, such as cation distribution, electronic structure and oxidation states, specific surface area, and particle size distribution, and the electrocatalytic performance for the OER in alkaline environment. Crucially, by decoupling the apparent observed catalytic activity from the intrinsic activity of the active sites, this work reveals how Cr and Mn distinctly influence the OER performance. By correlating these structural and electronic properties with the OER catalytic activity, this work aims to provide fundamental insights for the rational design of advanced spinel oxide electrocatalysts.

## 2 Materials and methods

### 2.1 Chemicals

For the synthesis of spinel oxides, sodium hydroxide (NaOH, ≥97.0%), tetraethylammonium hydroxide (TEAOH, 40% w/w in water), oxalic acid dihydrate (HO_2_CCO_2_H · 2H_2_O, ≥99.0%), nickel (II) nitrate hexahydrate (Ni(NO_3_)_2_ · 6H_2_O, ≥99.9%), iron (III) nitrate nonahydrate (Fe(NO_3_)_3_ · 9H_2_O, ≥98%), manganese (II) nitrate tetrahydrate (Mn(NO_3_)_2_ · 4H_2_O, ≥99.9%), chromium (III) nitrate nonahydrate (Cr(NO_3_)_3_ · 9H_2_O, 99%) were purchased from Merck. All reagents were used without further purification.

### 2.2 Synthesis

For the hydrothermal synthesis of a generic NiFe_2-X_M_X_O_4_ spinel oxide, a 11 mL suspension of metal oxalates was prepared under constant stirring from an aqueous solution of metal nitrates (total metal salts concentration of 0.225 M, with metal molar ratios corresponding to the target stoichiometry), and oxalic acid as chelating precipitant (with a concentration = $\left[M^{3+}\right]\times 1.5+\left[M^{2+}\right]$ ; where $\left[M^{3+}\right]$ is the total molar concentration of trivalent metal nitrates and $\left[M^{2+}\right]$ is the total molar concentration of divalent metal nitrates), in the presence of tetraethylammonium hydroxide (TEAOH) as a peptising agent (0.3 mL of 40% w/w aqueous solution added to the starting metals solution), and finally basified to pH ≈ 10 using a 10 M NaOH solution to deprotonate the oxalic acid causing the precipitation of the oxalates. The resulting suspension was then loaded in a stainless steel 4745 Parr autoclave (PTFE liner, volume 23 mL, filling ratio ∼50%) and heated at 135 °C for 12 h. The resulting solid powders were isolated by centrifugation, washed three times with deionised water and once with ethanol, and finally dried in vacuum at 80 °C overnight. The sample NiFeMnO_4_ was finally calcined at 500 °C for 2 h.

### 2.3 Characterisation methods

The elemental composition of the samples was determined by inductively-coupled plasma atomic emission spectroscopy (ICP-AES) using a Perkin Elmer Optima 4200 DV ICP-OES featuring a dual-view RF plasma source and a two-dimensional charge-coupled device array. A calibration curve was performed before each series of analyses. Before measuring, samples were digested in acidic environment.

Powder X-ray diffractograms (XRD) were acquired with a Bruker AXS D8 Advance Plus diffractometer, equipped with a Cu Kα_1,2_ anode (λ = 1.5106 1.5406 Å) and mounted with a LYNEXEYE XE-T detector employed in 1D mode. X-rays were generated by supplying a voltage of 40 kV and a current of 40 mA to the Cu anode, and data were collected with Bragg–Brentano geometry (2θ-θ). The diffractograms were recorded in the 2θ range 20°–80°, with a step size of 0.025° for 0.5 s/step. Fixed divergence slits of 0.50° were employed together with Soller slits with an aperture of 2.5°. A maximum position sensitive detector opening was used (2.83°). Pawley Refinement were carried out with Topas v7. The background was modelled with a Chebyshev polynomial function, and the peak profiles were fitted using a Double-Voigt approach to account for both crystallite size and lattice strain line broadening. The refined parameters included the background, sample displacement, the cubic lattice parameter *a*, crystallite size, and lattice strain.

The morphology and microstructure of the samples were characterised by transmission electron microscopy (TEM) and high-angle annular dark-field (HAADF) scanning transmission electron microscopy (HAADF-STEM) using a JEOL F200 microscope operated at 200 kV. Elemental analysis and mapping were performed using a JEOL 100 mm^2^ silicon drift energy dispersive X-ray spectrometer (EDX). Carbon-supported copper grids, 400 mesh size, were used for sample preparation.

N_2_ adsorption–desorption isotherms were recorded at −196 °C (77 K) using a Micromeritics ASAP 2020 Plus Instrument. Before the measurements, samples were degassed at 120 °C for 12 h under vacuum to ensure the complete removal of any previous adsorbate. The specific surface area (SSA) of the samples was calculated by a multipoint Brunauer–Emmett–Teller (BET) analysis in the 0.05–0.3 *p*/*p*_0_ range.

The analysis of the surface composition was carried out by X-ray photoelectron spectroscopy (XPS). The samples were analysed with a ThermoScientific Escalab QXi XPS spectrometer equipped with a monochromatic Al Kα source (hν = 1486.6 eV, full width at half maximum (FWHM) = 0.70 eV). Charge compensation was carried out by means of a dual-beam low-energy electron and ion coaxial flood source. The working pressure was 10^−7^ mbar. The survey spectra were acquired in the range 0–1350 eV (pass energy 200 eV, 1.0 eV/step, 100 ms/step); high resolution acquisitions (pass energy 50 eV, 0.05 eV/step, 50 ms/step) were performed for the regions of interest of the spectrum. Acquired spectra were analysed using the Avantage software (v. 6.9.4 Build 00001, Thermo Fisher Scientific), using the cross-section provided by the software for quantification of the species. The XPS spectra were analysed using the binding energies reported in the NIST XPS database as reference for peak assignment and chemical-state identification (Justin, 2012).

Raman spectra were collected by using a Thermo DXR Raman Microscope equipped with a 532 nm laser as excitation source operating at 10 mW power. The wavelength range was 150–1000 cm^−1^ recording 10 scans for each sample with an exposure time of 10 s.

The electrochemical features of the samples as electrocatalysts (ECs) for the oxygen evolution reaction (OER) were studied “*ex-situ*” in a conventional three-electrode cell mounting a graphite rod counter electrode and a Hg(l)|HgO(s)|KOH(aq) (0.1 M) reference electrode. The cell was filled with a 0.1 M KOH solution used as electrolyte. The measurements were collected at room temperature by means of a BioLogic SP-300 potentiostat/galvanostat run by the EC-Lab V11.43 software. The working electrode was prepared by drop-casting a catalyst ink, containing the spinel oxide, Vulcan™ XC72R carbon black, and Nafion dispersion, onto a glassy carbon rotating disk electrode (RDE), achieving a specific catalyst loading of 0.185 mg/cm^2^. Polarisation curves for the OER were recorded in Linear Sweep Voltammetry (LSV) mode at a scan rate of 5 mV/s (positive-going scans). All reported potentials refer to the reversible hydrogen electrode (RHE) scale, and the experimentally measured potentials were corrected for the ohmic drop corresponding to the uncompensated solution resistance (Ru), determined via impedance measurements. Detailed descriptions of the ink formulation, working electrode fabrication, reference electrode calibration, and electrochemical conditioning protocols are provided in the Supplementary Material.

## 3 Results and discussion

### 3.1 Synthesis and chemical-physical characterisations

**FIGURE 1:**
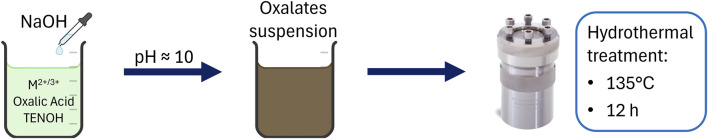
Synthetic scheme of the spinel oxides studied in the present project.

The materials were synthesised via a sub-critical hydrothermal treatment of a metal oxalates suspension (Diodati et al., 2014; Dolcet et al., 2018), as schematised in Figure 1. This specific synthetic route was selected since it was previously used to synthesise phase-pure spinel oxides nanoparticles with controlled morphology. The Fe substitution levels in NiFe_2-X_M_X_O_4_ (X = 0.25, 0.50, and 1.00) were specifically selected to systematically probe the evolution of the material properties across various doping degrees. This allowed us to observe the initial electronic modulations at X = 0.25, target an intermediate doping regime at X = 0.50, and investigate the solid solution limits and nanostructuring effects at an equimolar Fe:M ratio (X = 1.00).

In Table 1, the comparison of the desired stoichiometries with the experimental ones determined by ICP-OES analyses is reported.

**TABLE 1:** Expected and experimental stoichiometry of the synthetised samples. The experimental stoichiometry was calculated form ICP-OES results.

Expected stoichiometry	Experimental stoichiometry
NiFe_2_O_4_	Ni_0.98_Fe_2.02_O_X_
NiFe_1.75_Cr_0.25_O_4_	Ni_0.99_Fe_1.79_Cr_0.21_O_X_
NiFe_1.5_Cr_0.5_O_4_	Ni_1.04_Fe_1.54_Cr_0.43_O_X_
NiFeCrO_4_	Ni_1.04_Fe_1.07_Cr_0.90_O_X_
NiFe_1.75_Mn_0.25_O_4_	Ni_0.98_Fe_1.78_Mn_0.24_O_X_
NiFe_1.5_Mn_0.5_O_4_	Ni_0.94_Fe_1.42_Mn_0.64_O_X_
NiFeMnO_4_	Ni_0.93_Fe_1.09_Mn_0.99_O_X_

**FIGURE 2:**
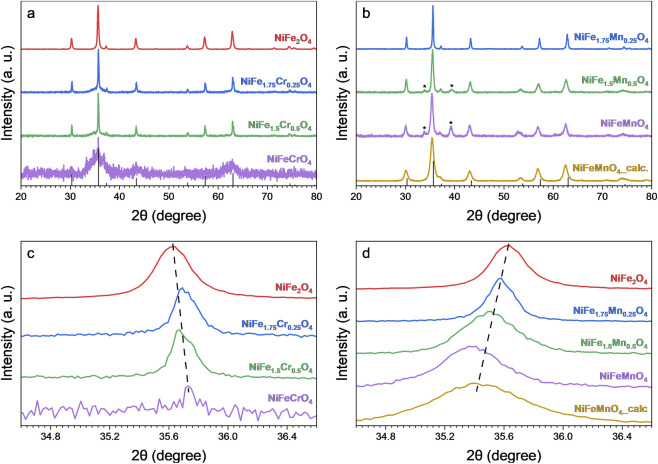
X-ray diffractograms of the: **(a)** Cr-containing samples, **(b)** Mn-containing samples. The asterisks indicate the reflections of a secondary phase composed of Ni(OH)_2_ (PDF 01-073-6992). **(c,d)** highlight the shift of the most intense reflection (311) for the Cr-containing and Mn-containing samples, respectively.

The ICP-OES results show a good agreement between the expected stoichiometries and the experimental ones for all the samples. Moreover, the XRD results confirm the spinel crystal structure of the sample, as all expected reflections typical of the spinel structure are observed in all the materials, confirming the successful syntheses of the targeted materials (Figure 2). Nevertheless, the diffractograms of the Mn-containing samples show that, by increasing the Fe-substitution degree (X = 0.5, and 1.0), a secondary crystalline phase is formed showing a diffraction pattern that corresponds to Ni(OH)_2_ (PDF 01-073-6992). This result indicates that the hydrothermal process alone, developed and optimised for bimetallic systems such as NiFe_2_O_4_ (Diodati et al., 2014), was not sufficient to achieve a complete inclusion of the three different metals in the same lattice.

The formation of this Ni(OH)_2_ secondary phase can be rationalised by the competitive nucleation kinetics and thermodynamic site preferences of the constituent cations. Recent comparative studies on hydrothermally synthesised spinel ferrites have demonstrated that the formation of the Mn-based spinel MnFe_2_O_4_ is kinetically favoured compared to the Ni-analogue NiFe_2_O_4_ under identical conditions (Andersen et al., 2025). This kinetic difference is linked to the electronic configuration of the divalent cations; the high-spin Mn^2+^ ion (d^5^) possesses zero crystal field stabilisation energy (CFSE), resulting in no strong preference for a specific crystallographic site. This flexibility allows Mn to more rapidly form a mixed or normal spinel structure without significant energy barriers. Conversely, the incorporation of Ni is more demanding. The Ni^2+^ ion (d^8^) exhibits a high CFSE in octahedral coordination, leading to a strong thermodynamic preference for the inverse spinel configuration (O'Neill, H. St. C., 1983; Szotek et al., 2006). The necessity for Ni^2+^ ions to specifically occupy octahedral sites creates a kinetic bottleneck compared to the faster integration of Mn. Consequently, at high substitution levels (X = 0.50, 1.00), the Mn-Fe spinel phase likely nucleates faster, consuming the available Fe^3+^ ions and competitively excluding the Ni^2+^ ions, which react slower to form the spinel structure. Due to the strongly basic reaction environment (pH ≈ 10), these “free” Ni^2+^ ions do not remain in solution but precipitate in the form of Ni(OH)_2_, forming the secondary phase observed in the diffractograms (Figure 2B) (Gerken et al., 2011; Wang et al., 2020)

To determine whether a subsequent thermal process was sufficient to induce the diffusion of the Ni cations of Ni(OH)_2_ into the spinel lattice, a calcination process was carried out at 500 °C for 2 h on the NiFeMnO_4_ sample. At the same time, high calcination temperatures (>500 °C) were excluded to avoid the risk of particle sintering and the consequent decrease in specific surface area. As demonstrated in Figure 2B, the diffraction reflections associated with the Ni(OH)_2_ phase are absent in the calcined sample (NiFeMnO_4__calc, Figure 2B), indicating the decomposition of the Ni hydroxide.

It is worth mentioning that attempts were also made to obtain a phase pure NiFeMnO_4_ composition directly via hydrothermal synthesis by increasing the treatment temperature to 250 °C, still for 12 h. However, this approach also proved to be unsuccessful since XRD analyses revealed a notable content of crystalline carbonates, the diffraction pattern of which corresponds to MnCO_3_ (COD 2101387), along with the spinel structure (Supplementary Figure S1). The presence of carbonates likely originates from the decomposition of the oxalate precursors into CO_2_ at elevated temperatures, which, in the strongly basic reaction environment, reacted with the dissolved metal cations to form insoluble carbonates. Therefore, the lower temperature synthesis followed by calcination was selected as the most effective strategy for the synthesis of the spinel oxide with composition NiFeMnO_4_.

The crystallite sizes of the synthesised materials, calculated using Scherrer’s equation (with parameters detailed in Supplementary Table S1), are reported in Table 2. The Mn-doped samples show a general trend of decreasing crystallite size upon substitution, dropping from 29 nm for the pristine NiFe_2_O_4_ down to 20 nm for NiFe_1.75_Mn_0.25_O_4_ and NiFe_1.5_Mn_0.5_O_4_, and 12 nm for NiFeMnO_4_. The sample NiFeMnO_4__calc exhibits crystallites with mean diameter of 16 nm, larger than those of the not calcined sample, indicating a possible partial sintering of the crystallites.

**TABLE 2:** Crystallite size for the as-synthetised samples calculated applying the Scherrer’s equation (Patterson, 1939).

Sample	Crystallite (nm)
NiFe_2_O_4_	29
NiFe_1.75_Cr_0.25_O_4_	44
NiFe_1.5_Cr_0.5_O_4_	65
NiFeCrO_4_	3
NiFe_1.75_Mn_0.25_O_4_	20
NiFe_1.5_Mn_0.5_O_4_	20
NiFeMnO_4_	12
NiFeMnO_4__calc	16

Conversely, the Cr-doped series exhibits a non-linear behaviour. At partial substitution levels (NiFe_1.75_Cr_0.25_O_4_ and NiFe_1.5_Cr_0.5_O_4_), a narrowing of the main diffraction peaks is observed, which corresponds to an apparent increase in crystallite size to 44 nm and 65 nm, respectively. However, a distinct broad “bump” is noticeable at the base of the sharp (311) reflection in their diffractograms. This feature is indicative of a bimodal size distribution, suggesting the co-existence of larger, highly crystalline domains alongside a substantial fraction of very small nanoparticles or an amorphous phase. Eventually, at complete substitution, the sample NiFeCrO_4_ displays a dramatic broadening of the diffraction peaks, reflecting a drastic reduction in crystallite size down to 3 nm. The overall reduction in crystallite size observed for the Mn-series and for the fully substituted NiFeCrO_4_ sample can be ascribed to the lattice distortion and internal strain arising from the mismatch in ionic radii and electronic configurations between the host cations and the dopants (Dippong et al., 2022; Nazari et al., 2024; Rui et al., 2025). In the case of partial Cr substitution, this structural disorder likely promotes the observed bimodal distribution, hindering the uniform crystal growth. The incorporation of these transition metals introduces structural disorder, which acts as an energy barrier hindering crystal growth. Specifically regarding the Cr-doped samples, recent theoretical investigations suggest that the lattice mismatch and the resulting stress at the grain growth interface can effectively suppress subsequent crystal growth (Rui et al., 2025). Furthermore, the variation in metal-oxygen bond energies and the alteration of cation distribution upon doping can modify the nucleation and growth kinetics, favouring the formation of smaller crystallites (Nazari et al., 2024).

Additionally, the XRD patterns reveal a slight shift in the diffraction reflections depending on the dopant, particularly evident for the most intense (311) reflection (Figures 2C,D). In Cr-substituted samples, the reflections shift toward higher angles with respect to the pattern of NiFe_2_O_4_ (PDF 49-0188), indicating a lattice contraction as a consequence of the incorporation of smaller cations. This shift confirms the incorporation of Cr^3+^ ions (ionic radius: 0.615 Å, O_h_) into the spinel lattice, likely in O_h_ sites for their CFSE ( $t_{2g}^{3}e_{g}^{0}$ , $CFSE=-6/5{\;\Delta }_{0}$ ), as they have a smaller ionic radius than Fe^3+^ (0.645 Å in O_h_). In contrast, Mn-substituted samples exhibit a shift in diffraction peaks toward lower angles, indicating a lattice expansion. From XPS results, Mn is primarily present as Mn^2+^; however, the co-existence of Mn^3+^ species cannot be excluded. The lattice expansion is attributed to the incorporation of Mn^2+^ (0.66 Å in T_d_ and 0.83 Å in O_h_, (Shannon, 1976), larger than Fe^3+^ (0.49 Å in T_d_, and 0.645 Å in O_h_) whereas the latter is considered to occupy both T_d_ and O_h_ sites for its CFSE equal to zero (d^5^, $t_{2g}^{3}e_{g}^{2}$ , $CFSE=0{\;\Delta }_{0}$ ). The Mn^3+^ ions would not cause such expansion: Mn^3+^ (d^4^, favoured in O_h_ sites for its $CFSE=-3/5\;\Delta_{0}$ , 0.645 Å) is identical in size to Fe^3+^ (0.645 Å in O_h_). As for the Cr-doped samples, also in the case of the Mn-doped samples the observed shifts of the (311) reflection follow an expected trend, confirming the incorporation of the Mn cations in the spinel lattice. The data of the ionic radii are reported by Shannon, 1976.

To quantitatively support these observations, a Pawley refinement of the XRD patterns was performed for the pristine NiFe_2_O_4_, the fully Cr-substituted sample (NiFeCrO_4_), and the fully Mn-substituted sample (NiFeMnO_4__calc). The corresponding fitting curves are reported in the (Supplementary Figures S2–4). The results are reported in Table 3. The refinement results confirm a lattice contraction upon Cr doping, with the lattice parameter decreasing from 8.36 Å for the pristine NiFe_2_O_4_ down to 8.31 Å for NiFeCrO_4_. Conversely, the incorporation of Mn leads to a lattice expansion, with the parameter increasing to 8.41 Å for NiFeMnO_4__calc. These variations in the unit cell dimensions perfectly reflect the differences in ionic radii between the Fe^3+^ and dopant cations, successfully confirming the incorporation of Cr^3+^ and Mn^2+^ into the spinel lattice. Furthermore, the average crystallite sizes derived from the refinement (26.5 nm for NiFe_2_O_4_, 3.0 nm for NiFeCrO_4_, and 8.2 nm for NiFeMnO_4__calc) are in agreement with the structural trends previously observed using Scherrer’s equation (Table 2).

**TABLE 3:** Structural parameters (average crystallite size and lattice parameter *a*) calculated via Pawley refinement of the X-ray diffractograms for the samples NiFe_2_O_4_, NiFeCrO_4_, and NiFeMnO_4__calc.

Sample	Average crystallite size (nm)	Lattice parameter (Å)
NiFe_2_O_4_	26.5	8.36
NiFeCrO_4_	3.0	8.31
NiFeMnO_4__calc	8.2	8.41

Transmission electron microscopy (TEM) analyses were performed to study the morphology and size distribution of the synthesised nanoparticles. These analyses were combined with Selected Area Electron diffraction (SAED) for investigating the crystal structure at the nanoscale, and energy-dispersive X-ray spectroscopy (EDX) for elemental mapping. The analysed samples were NiFe_2_O_4_, NiFe_1.75_Cr_0.25_O_4_, NiFeCrO_4_, NiFe_1.75_Mn_0.25_O_4_, and NiFeMnO_4__calc. It is important to point out that the preparation of the TEM specimens via the drop-casting of the particles suspension onto the grid resulted in the formation of clusters composed of agglomerated and overlapping nanoparticles. This phenomenon inevitably introduces a degree of uncertainty in the precise quantification of the individual particle dimensions, as the boundaries of the overlapping particles are not always clearly distinguishable. Consequently, the particle size estimation presented herein should be interpreted primarily as an assessment of the mean diameter trends induced by the doping, rather than an absolute determination of the geometric mean diameter.

**FIGURE 3:**
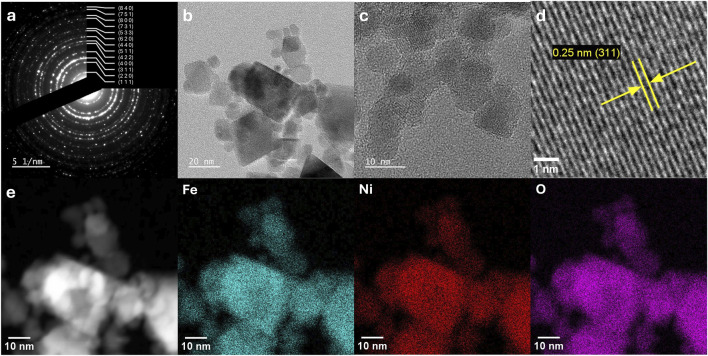
TEM, SAED, and EDX results of the sample NiFe_2_O_4_: **(a)** SAED pattern, **(b–d)** TEM micrographs, and **(e)** EDX elemental mapping.

**FIGURE 4:**
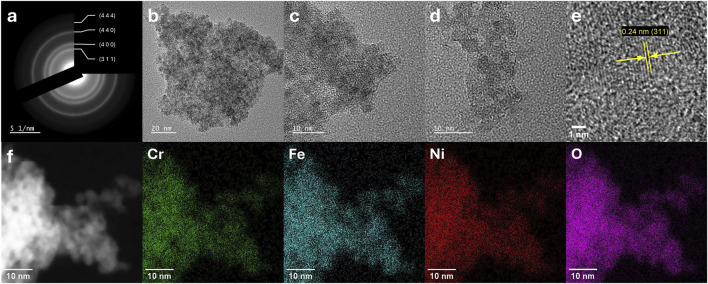
TEM, SAED, and EDX results of the sample NiFeCrO_4_: **(a)** SAED pattern, **(b–e)** TEM micrographs, and **(f)** EDX elemental mapping.

**FIGURE 5:**
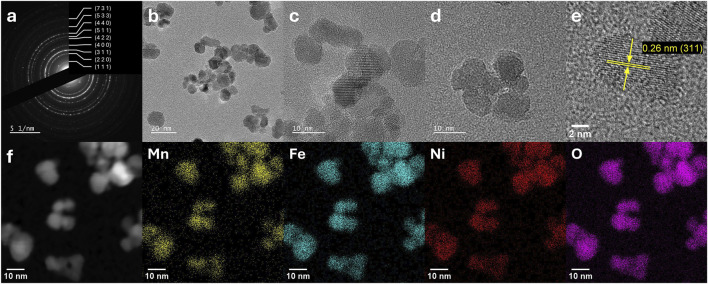
TEM, SAED, and EDX results of the sample NiFeMnO_4__calc: **(a)** SAED pattern, **(b–e)** TEM micrographs, and **(f)** EDX elemental mapping.

The presence of the lattice fringes in TEM micrographs confirms that all the materials are crystalline (Figures 3–5; Supplementary Figures S6, S9). As reported in Figure 3, the sample NiFe_2_O_4_ is composed of the largest particles and exhibits the broadest size distribution (15.7 ± 10.6 nm). As anticipated by the XRD analysis (Table 2), the samples exhibit significant morphological variations upon doping, generally leading to the formation of smaller nanoparticles. The TEM micrographs of the sample NiFe_1.75_Mn_0.25_O_4_ (Supplementary Figure S9), show that the material is composed of nanoparticles with a smaller mean diameter than the pristine sample NiFe_2_O_4_ (13.9 ± 6.1 nm). Increasing the Fe substitution degree with Mn, the sample NiFeMnO_4__calc (Figure 5) displays particles with a mean diameter of 8.4 ± 3.6 nm, confirming again the trend observed for the average crystallite size determined by XRD analyses (Table 2). The limitation on particle-size determination caused by overlapping particles is particularly pronounced in the Cr-containing samples, where the nanoparticles strongly tend to form aggregates; consequently, a precise statistical estimate of the mean diameter would be highly imprecise. Nevertheless, a qualitative analysis of the TEM micrographs allows for clear morphological distinctions. Interestingly, while XRD calculated an apparent crystallite size of 44.3 nm for NiFe_1.75_Cr_0.25_O_4_ due to the sharp diffraction peak, the TEM images (Supplementary Figure S6) reveal that the material is predominantly composed of much smaller nanoparticles approximately in the 2–8 nm range. This finding perfectly corroborates the bimodal size distribution hypothesised from the “bump” observed in the diffractogram: the bulk of the material consists of very small nanoparticles, while a minor fraction of larger, highly crystalline domains (not observed in TEM) dominates the coherent scattering in the XRD pattern. Finally, the highly Fe-substituted NiFeCrO_4_ (Figure 4) is homogeneously composed of even smaller crystalline particles (<5 nm), consistently matching the drastic crystallite size reduction (3 nm) calculated from XRD data (Table 2). Moreover, in the latter case for the sample NiFeCrO_4_, the presence of crystalline particles with diameter <5 nm also explains the absence of any intense and sharp reflection in the X-ray diffractogram (Figure 2A, NiFeCrO_4_) (Cullity and Stock, 2001; Holder and Schaak, 2019; Langford and Wilson, 1978; Patterson, 1939)

SAED is a very powerful technique for studying the nanoscale crystallography and phase purity of these materials. SAED patterns were acquired from multiple regions to ensure the results were representative of the entire sample. For all samples, the SAED patterns exhibit diffraction rings indexed to the cubic spinel lattice. No additional sets of rings or discrete spots attributable to secondary crystalline phases are observed (Figures 3–5; Supplementary Figures S6, S9). For NiFeCrO_4_, despite the very small particle size, SAED reveals weak diffraction rings but consistent with the planes (311), (400), (440), and (444) of the spinel structure, which are the most intense reflections in the XRD. For the samples NiFe_2_O_4_, NiFe_1.75_Mn_0.25_O_4_, and NiFeMnO_4__calc, SAED patterns collected from larger particles show well defined diffraction rings, indicating the presence of high crystalline structures having the expected spinel oxide structure (Figures 3, 5; Supplementary Figure S9

Complementary to the structural analysis, EDX spectroscopy integrates the structural information by mapping the distribution of the metal elements at nanometer resolution. With the notable exception of the calcined Mn-doped sample, the recorded maps show the spatial overlap of the signals of all elements suggesting a generally uniform composition rather than the formation of segregated domains (see elemental mapping on Figures 3, 4; Supplementary Figures S5–11; Supplementary Figures S12–16 for the EDX spectra). Even for the fully substituted Cr-doped sample, NiFeCrO_4_, no large-scale enrichment of any metal at grain boundaries or particle surfaces could be observed. As shown in Table 4, the elemental composition derived from the EDX spectra confirms a good agreement with the expected stoichiometry for the samples NiFe_2_O_4_, NiFe_1.75_Cr_0.25_O_4_, NiFeCrO_4_, and NiFe_1.75_Mn_0.25_O_4_. For the pristine sample NiFe_2_O_4_, the atomic ratio Ni: Fe was calculated to be 1.01 : 1.99, which is virtually identical to the theoretical value of 1.00 : 2.00, confirming the expected stoichiometry of the material. Similarly, the introduction of dopants, both for Cr and Mn, at lower concentrations (X = 0.25) resulted in a calculated elemental composition in agreement with the targeted stoichiometries. The sample NiFe_1.75_Cr_0.25_O_4_ exhibited a Cr: Fe: Ni ratio of 0.26: 1.70: 1.05, closely matching the expected value of 0.25: 1.75: 1.00. In the case of NiFe_1.75_Mn_0.25_O_4_, the calculated experimental ratio Mn: Fe: Ni is 0.21: 1.76: 1.03, which confirms the successful synthesis of the targeted composition with ratio 0.25: 1.75: 1.00. Increasing the Fe substitution to X = 1 with Cr, the sample NiFeCrO_4_ shows an experimental Ni: Fe: Cr ratio of 1.02:1.01:0.97, very close to the target 1: 1: 1. However, when Fe is substituted with Mn with a substitution degree of X = 1, the experimental elemental composition of the sample NiFeMnO_4__calc presents a notable discrepancy compared to the expected value. In contrast to the ICP result (Table 1), which represents the average bulk composition and returns an elemental composition which matches the 1: 1: 1 target stoichiometry, the local EDX analyses provide a stoichiometric ratio of Mn: Fe: Ni equals to 0.68 : 1.73: 0.59. This reveals a significant enrichment in Fe and a depletion in both Ni and Mn at the nanoscale. This result could indicate that the nanoscale composition of the sample NiFeMnO_4_ is not homogeneous, and the presence of spurious phases enriched in Ni and Mn, like NiMn_2_O_4_ and/or NiO, cannot be excluded. The latter could be formed from the Ni(OH)_2_ phase during the calcination in air of the as-synthesised sample NiFeMnO_4_. This hypothesis is further confirmed by the overlapping of the elemental maps of the NiFeMnO_4__calc sample (Supplementary Figure 11), which reveals a relatively higher concentration of Ni towards the surface of the particles. This surface accumulation suggests that the thermal treatment at 500 °C induces an incomplete solid-state diffusion of the Ni cations, originally precipitated as a separate Ni(OH)_2_ phase, into the bulk of the Mn-Fe-rich spinel crystals. The presence of these secondary phases is plausible also because they have a diffraction pattern similar to the targeted spinel material (cubic crystal system), making their detection particularly challenging by XRD and SAED. This also indicates that the calcination step of NiFeMnO_4_ after the hydrothermal treatment is not sufficient to obtain a single-phase material. Collectively, these results demonstrate that the hydrothermal method used enabled the achievement of an excellent compositional control and homogeneity even at the nanometre level, mirroring the bulk average provided by the ICP analyses, with the only exception of the sample NiFeMnO_4_.

**TABLE 4:** Elemental atomic percentages and stoichiometric ratios obtained from the EDX analyses. The %_at._ O are not reported because influenced by O-containing species adsorbed on the material surface. The expected composition NiFeMnO_4_ refers to the NiFeMnO_4__calc sample.

Expected composition	Atomic percentage (EDX)				Stoichiometric ratio (EDX)			
	%_at._ Cr	%_at._ Mn	%_at._ Fe	%_at._ Ni	Cr	Mn	Fe	Ni
NiFe_2_O_4_	–	–	20.69	10.47	–	–	1.99	1.01
NiFe_1.75_Cr_0.25_O_4_	2.12	–	13.96	8.61	0.26	–	1.70	1.05
NiFeCrO_4_	9.06	–	9.41	9.56	0.97	–	1.01	1.02
NiFe_1.75_Mn_0.25_O_4_	–	2.1	17.89	10.46	–	0.21	1.76	1.03
NiFeMnO_4_	–	5.32	15.74	6.19	–	0.59	1.73	0.68

To evaluate the specific surface area (SSA) of the synthesised samples, nitrogen physisorption measurements were conducted using the Brunauer–Emmett–Teller (BET) method (Table 5) (Brunauer et al., 1938) The SSA is an important parameter to evaluate, as a high SSA could be correlated with a higher surface density of accessible active sites for the catalytic reaction.

**TABLE 5:** Specific surface area of the samples evaluated with the BET method.

Sample	Specific surface area (m^2^/g)
NiFe_2_O_4_	39
NiFe_1.75_Cr_0.25_O_4_	191
NiFeCrO_4_	226
NiFe_1.75_Mn_0.25_O_4_	67
NiFeMnO_4_	100
NiFeMnO_4__calc	84

The SSA of the samples increases significantly by increasing the amount of Fe substitution, as shown in Table 5. This trend is particularly evident in the Cr-containing samples, where the SSA increases from 39 m^2^/g for the pristine NiFe_2_O_4_ to 191 m^2^/g for NiFe_1.75_Cr_0.25_O_4_, and reaches the higher value of 226 m^2^/g for NiFeCrO_4_. The Mn-doped samples also show a marked increase (NiFe_1.75_Mn_0.25_O_4_: 67 m^2^/g; NiFe_1.75_Mn_0.25_O_4_: 100 m^2^/g) compared to NiFe_2_O_4_, though less pronounced than the Cr-series. This trend correlates directly with the reduction of particle size observed by TEM (Figures 3–5, Supplementary Figures S6, S9). For the partially substituted Cr-doped samples (NiFe_1.75_Cr_0.25_O_4_ and NiFe_1.5_Cr_0.5_O_4_), the high SSA further supports the fact that the actual dominant morphological feature consists of small nanoparticles, despite the large apparent crystallite size derived from the sharp XRD reflections (Table 2). For instance, the NiFeCrO_4_​ sample, with its very small crystallites (3 nm) and particle size, exhibits the highest SSA of the entire series. This correlation between an increased degree of Fe substitution, decreased particle size, and higher SSA suggests that a considerable benefit of the doping strategy could lie not solely in an improvement of the intrinsic electronic properties, but also in an increase in the total number of available active surface sites. The lower SSA of the sample NiFeMnO_4__calc, compared to the sample before calcination, could be correlated with the removal of adsorbed organic residues of the synthetic process and to a partial sintering of the nanoparticles (Table 5).

**FIGURE 6:**
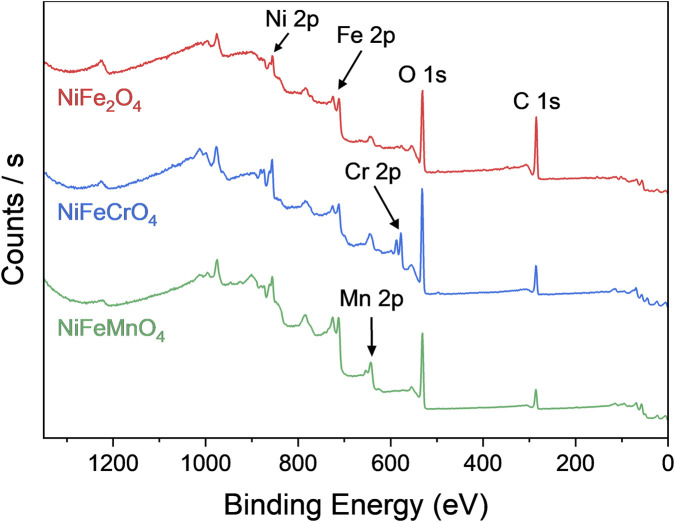
XPS survey spectra of the sample NiFe_2_O_4_, NiFeCrO_4_, and NiFeMnO_4__calc. The labels indicate the regions used for high-resolution spectral acquisition.

XPS analyses were conducted to investigate the surface chemical composition and oxidation states of the metals. Since the OER process occurs at the active material–electrolyte interface, the catalytic activity is intrinsically governed by surface properties. Survey scans (Figure 6, for the sample NiFe_2_O_4_, NiFeCrO_4_, and NiFeMnO_4__calc) confirmed the presence of all constituent elements on the material surfaces; however, an accurate and reliable quantitative analysis was hindered by significant spectral overlap of the different signals, including Auger signals (a typical situation when analysing oxides composed of multiple first row transition metals). Consequently, the discussion herein relies merely on a qualitative assessment of the chemical species. The high resolution XPS spectra and the correspondent deconvolutions are reported in Supplementary Figures S17–21.

This spectral overlap is particularly critical for the Mn 2p signal, which is strongly superimposed on the Ni LMM Auger line (when using an Al-K_α_ source; the involved Auger line is Ni L_3_M_23_M_45_). To isolate the Mn 2p contribution in the 700–745 eV region (Ansell et al., 1978), the following procedure was adopted: first, the Ni 2p_3/2_: Ni LMM area ratio was determined from a Mn-free sample, resulting in a value of ca. 1.9. This ratio was then constrained (within a 5% margin) during the fitting of Mn-containing samples, allowing the residual area to be attributed to the Mn 2p signal.

For all samples, the analysis of the Fe 2p region reveals a spectral shape consistent with that reported by Biesinger et al. for NiFe_2_O_4_ (Biesinger et al., 2011), confirming the presence of Fe^3+^ species in all samples. Also the Ni 2p region was fitted according to the NiFe_2_O_4_ model (Biesinger et al., 2011), identifying Ni^2+^ as the dominant species. Nevertheless, the more symmetric peak shape with respect to that reported for NiFe_2_O_4_ hints that the presence of Ni^3+^ (e.g., NiOOH) cannot be excluded due to the strong spectral similarity between Ni^2+^ in oxides and Ni^3+^ in oxyhydroxides as noted by Grosvenor et al. (2006) This consideration is particularly relevant considering that surface Ni^3+^ species, in the form of NiOOH, is widely considered to be one of the primary active sites for the OER (Limpt et al., 2023; Samarai et al., 2019; Gupta et al., 2015; Wang et al., 2015; Trotochaud et al., 2014; Hatab et al., 2022; Dubai et al., 2024).

Regarding the dopants, the Cr 2p region of the Cr-doped samples indicates the presence of Cr^3+^ (fitted as Cr_2_O_3_), alongside Cr^6+^ species identified via the NIST database (Justin, 2012). It is hypothesised that these Cr^6+^ species are concentrated primarily at the surface. Due to their high charge density and significantly smaller ionic radius compared to the host cations (Shannon, 1976), their incorporation into the bulk spinel lattice is considered to be energetically unfavourable.

Finally, for the Mn-doped samples, the Mn 2p spectra exhibit a broad shake-up satellite characteristic of Mn^2+^ (Biesinger et al., 2011); yet the position of the main peak maximum and the overall band shape resemble that found for MnOOH, suggesting the possible co-existence of Mn^3+^ species (Biesinger et al., 2011).

**FIGURE 7:**
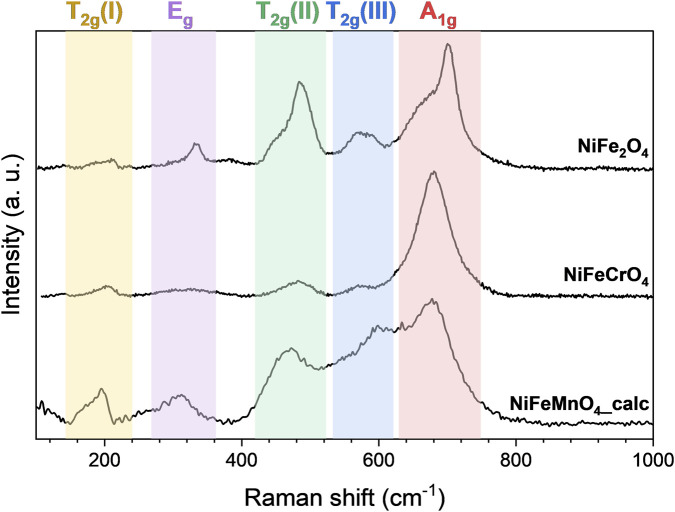
Raman spectra of the samples: NiFe_2_O_4_, NiFeCrO_4_, and NiFeMnO_4__calc. The shaded regions highlight the characteristic regions of vibrational modes of the spinel structure.

Spinel oxides crystallise in the cubic space group Fd3̅m, for which group theory predicts five first-order Raman-active phonon modes: one A_1g_ mode, one, E_g_ mode, and three T_2g_ modes (White and DeAngelis, 1967). These modes correspond to different vibrational motions within the spinel structure, specifically related to the metal-oxygen (M−O) bonds in the T_d_ and O_h_ sites. The A_1g_ mode, observed at approximately 690 cm^-1^ in NiFe_2_O_4_ (Figure 7), is attributed to the symmetric stretching of the M−O bonds in the tetrahedral site. The E_g_ mode, around 320 cm^-1^, is associated with symmetric bending of M−O bonds in the octahedral site. The three T_2g_ modes correspond to different vibrational motions in the octahedral sites: T_2g_ (1) at 180 cm^-1^ is due to translational movement of the tetrahedron, T_2g_ (2) around 470 cm^-1^ is linked to asymmetric stretching, and T_2g_ (3) near 570 cm^-1^ corresponds to asymmetric bending of the M−O bonds (Sanchez-Lievanos et al., 2021; D’Ippolito et al., 2015). The characteristic bands for Raman active vibrations in the spinel structure depend on the degree of inversion, since occupation of one crystal site by more than one different cation will result in a splitting of the observed modes (Sanchez-Lievanos et al., 2021). In many cases, the actual structure is somewhere in between the completely normal and completely inverted structure. While an accurate determination of the degree of inversion based on Raman measurements is difficult due to small differences in the observed Raman shifts and significant absorption of the incident laser light (532 nm) by the dark-brown/black samples, a qualitative assessment of the cation distribution might be attempted. As a proof of concept, the spectra of the samples NiFe_2_O_4_, NiFeCrO_4_ and NiFeMnO_4_ are reported in Figure 7.

The Raman spectrum of NiFe_2_O_4_ corresponds well with that reported in the literature (Sanchez-Lievanos et al., 2021; D’Ippolito et al., 2015; Lazarević et al., 2013). The spectrum displays broadened peaks with shoulders, which is an indication of the partially inverted spinel structure where both T_d_ and O_h_ sites are occupied by more than one cation (D’Ippolito et al., 2015; Lazarević et al., 2013; Shanigaram et al., 2022). The Raman spectrum of NiFeCrO_4_ displays a distinct change in the A_1g_ mode (related to the T_d_ site), which exhibits a higher relative intensity and a less pronounced shoulder compared to NiFe_2_O_4_. This spectral evolution suggests a more uniform cation distribution within the tetrahedral sites of the lattice. This observation aligns with the surface chemistry probed by XPS, specifically the confirmation of Cr^3+^ species. Due to its d^3^ electronic configuration ( $t_{2g}^{3}e_{g}^{0}$ , $CFSE=-6/5{\;\Delta }_{0}$ ) Cr^3+^ possesses a strong thermodynamic preference for octahedral (O_h_) sites. Consequently, compared to NiFe_2_O_4_, it is hypothesised that the incorporation of Cr^3+^ displaces Fe^3+^ cations from the O_h_ sites, forcing them to occupy the T_d_ sites. This redistribution results in a T_d_ coordination populated predominantly by Fe^3+^ cations; the resulting homogeneity of the Fe–O (T_d_) vibrational modes explains the increased symmetry and reduced broadening of the A_1g_ signal compared to the more complex T_d_ occupation in NiFe_2_O_4_. As expected, the broad width of the, E_g_, T_2g_ (2) and T_2g_ (3) signals suggest a more complex occupation of the O_h_ sites, likely originating from the vibration of the Ni−O (O_h_) and Cr−O (O_h_) bonds. The Raman spectrum of NiFeMnO_4_ is characterised by broad and strongly overlapping signals, reflecting a highly disordered crystal structure. This observation aligns with the chemical complexity identified by XPS, which indicates the presence of Mn^2+^ alongside Fe^3+^. Significantly, both high-spin Mn^2+^ and Fe^3+^ cations possess a d^5^ ( $t_{2g}^{3}e_{g}^{2}$ ) electronic configuration; consequently, they have a CFSE equal to zero, meaning they lack a strong thermodynamic preference for either T_d_ or octahedral O_h_ coordination. This fact promotes a random distribution of these cations across both T_d_ and O_h_ sub-lattices. Consequently, the presence of M–O bonds in both O_h_ and T_d_ coordinations involving multiple metallic species results in the broadening and overlapping of the observed Raman bands.

### 3.2 Electrochemical tests

**FIGURE 8:**
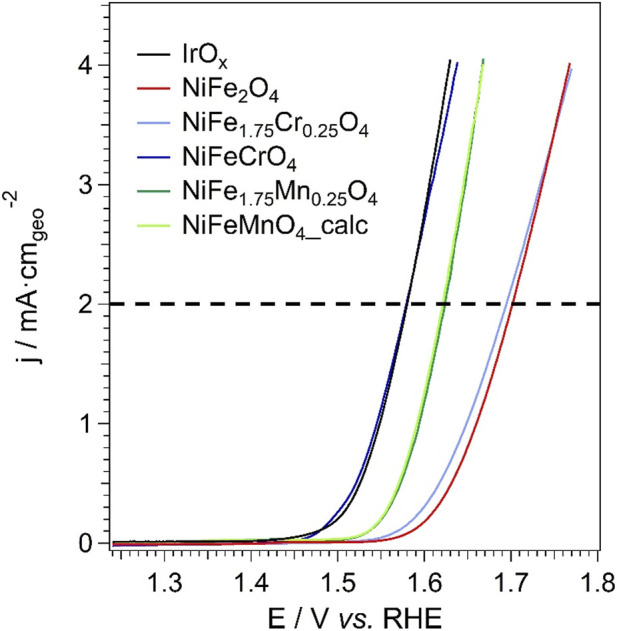
LSV polarization curves of the spinel oxide samples.

The electrocatalytic efficiency of the synthesised materials toward the OER was assessed using Linear Sweep Voltammetry (LSV). These curves are instrumental in quantifying the OER overpotential ( η , defined as the difference between the applied potential and the thermodynamic value of 1.23 V) required to achieve a specific geometric current density relative to the geometrical surface area of the rotating disk electrode (RDE). The overpotential serves as a figure of merit for catalytic activity; a lower value corresponds to superior performance, indicating that a good OER current density can be obtained by a low polarisation of the working RDE. The analysed samples have composition NiFe_2-X_M_X_O_4_ (M: Cr, Mn; X: 0.25, 1.00), including the reference NiFe_2_O_4_ and the benchmark IrO_X_. This choice was made for clarity and conciseness, focusing on the compositions that most effectively highlight the structure–property relationships within the investigated series. It is important to note that the as-synthesised sample with the stoichiometry NiFeMnO_4_ was not included in the LSV tests due to the presence of the secondary phase. Hence, this composition was investigated using the sample obtained after the calcination at 500 °C for 2 h. In Figure 8 the LSV polarisation curves are reported. The measured current is normalised by the geometric area of the RDE. The overpotential of the various samples is evaluated based on the potential recorded at 2 mA·cm_geo_^−2^ as a figure of merit of the intrinsic electrocatalytic behaviour of the electrocatalysts discussed in this work. The comparison between the OER overpotentials of the electrocatalysts was carried out at 2 mA·cm_geo_^−2^ to better probe the fundamental kinetic regime while minimizing contributions from mass-transport limitations, bubble coverage, and other secondary effects that become increasingly relevant at higher current densities (Di Noto, 2024). It is highlighted that this study is primarily focused on elucidating structure–property relationships and intrinsic electrochemical trends within the investigated spinel series, rather than on device-level benchmarking under industrially relevant current densities.

**FIGURE 9:**
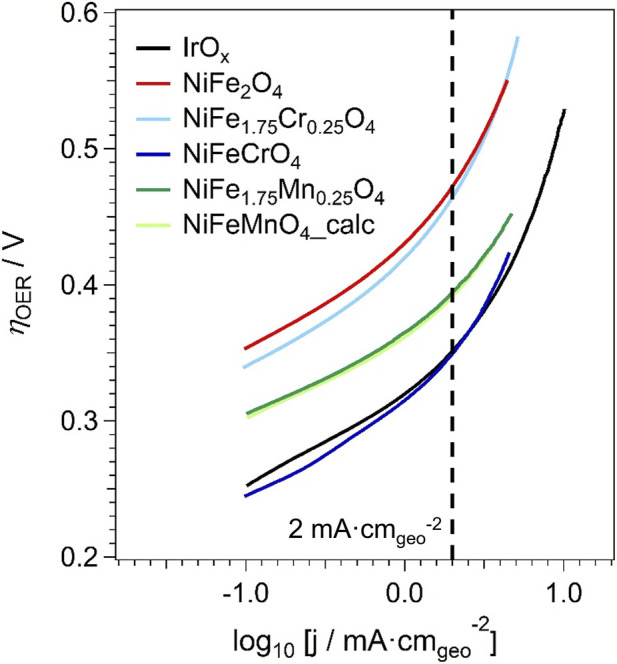
Tafel plots of the LSV curves for the OER.

The data displayed in Figure 8 can be retraced in a semilogarithmic scale, yielding the Tafel plot (Bard et al., 2022; Heijden et al., 2024). The data are plotted in Figure 9 as a function of the overpotential for the OER (*η*_OER_). A closer inspection of the Tafel plots (Figure 9) reveals two distinct regimes. At low geometric current densities (log_10_ [j/mA·cm_geo_^−2^] < −0.5), the Tafel slope assumes values in the range 0.052–0.066 V·dec^−1^. At higher current densities, the slope progressively increases. Such behavior is commonly observed for the OER in alkaline media and has been widely reported in the literature (Heijden et al., 2023; Liu et al., 2023). The increase of the apparent Tafel slope at higher current densities is typically associated with a transition from a purely kinetic regime to a mixed kinetic-mass transport regime and/or to a change in the rate-determining step under increasing polarisation. Similar trends are observed not only for the spinel oxides investigated in this work but also for the IrO_x_ benchmark electrocatalyst.

The comparison of the OER performance of the samples must also account for the different number of active sites exhibited by each sample. In this study, the Electrochemically Active Surface Area (ECSA) was approximated using the BET specific surface area, a common method for comparing intrinsic activities of materials within the same class (Esswein et al., 2009; Fabbri et al., 2014; He et al., 2025b; McCrory et al., 2013). This approach assumes that the majority of the physical surface area is electrochemically accessible. Thus, the ECSA of each sample was evaluated according to Equation 1:

$$\text{ECSA }\left({\text{cm}}^{2}\right)=\text{SSA }\left(m^{2}\cdot g^{‐1}\right)\times \text{mass of the spinel on the RDE tip }\left(\mu g\right)$$ (1)

**FIGURE 10:**
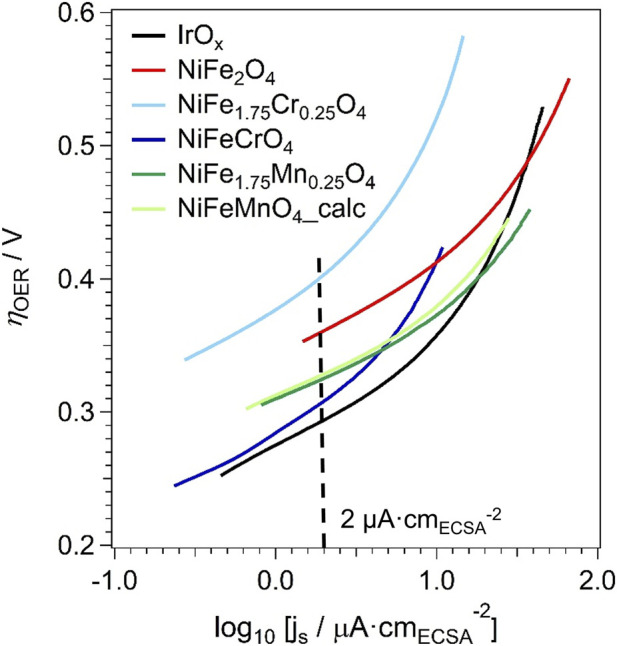
Tafel plots of the specific current densities (j_s_) in the OER.

The SSA is determined by N_2_ physisorption measurements (Table 5); the mass of the sample on the RDE tip is equal to 43.8 μg for all the tests. To compare the OER performance across different materials, the OER currents are normalised by the corresponding ECSA, yielding the *“specific current density”* (*j*_s_), expressed in μA·cm_ECSA_^-2^. The results are reported in Figure 10. To facilitate the comparison between the OER performance of different samples, their *η*_OER_ is compared at the same *j*_s_ value, 2 μA·cm_ECSA_^-2^. The value of the Tafel slope in the OER, *β*_OER_, is also determined at the same *j*_*s*_ for all the samples. *β*_OER_ is used to study the OER mechanism and it is listed in Table 6.

**TABLE 6:** Figures of merit describing the OER performance of the samples.

Sample	SSA (m^2^·g^-1^)	ECSA (cm^2^)	$\eta_{OER}$ @ j = 2 $mA\cdot {cm}_{geo}^{-2}$ (mV)	$\eta_{OER}$ @ j_s_ = 2 $\mu A\cdot {cm}_{ECSA}^{-2}$ (mV)	$\beta_{OER}$ @ j = 2 $\mu A\cdot {cm}_{ECSA}^{-2}$ (mV·dec^−1^)	$j_{0,OER}$ (m $A\cdot {cm}_{ECSA}^{-2}$ )
NiFe_2_O_4_	39	15.8	472	361	171.2	10^-(8.31±0.02)^
NiFe_1.75_Cr_0.25_O_4_	191	83.7	465	403	186.2	10^-(8.81±0.04)^
NiFeCrO_4_	226	99.1	349	307	146.1	10^-(7.82±0.07)^
NiFe_1.75_Mn_0.25_O_4_	67	29.38	394	325	124.9	10^-(8.93±0.03)^
NiFeMnO_4__calc	84	36.83	391	329	121.5	10^-(8.82±0.02)^
IrO_X_	120^*^	52.6	351	294	129.5	10^-(7.18±0.03)^

^*^ Nominal value.

In order to determine the exchange current density (*j*_0,OER_), linear fitting was performed in the low-current-density region, where the Tafel slope remains in the 0.052–0.065 V·dec^−1^ range and mass-transport contributions can reasonably be considered negligible. The corresponding linear fits and extrapolations to *η*_OER_ = 0 (Di Noto et al., 2021; 2021) are reported in the (Supplementary Figure S22). The uncertainty associated with the determination of *j*_0,OER_ was estimated by propagating the uncertainties of the slope and intercept obtained from the linear Tafel fits. The detailed procedure and the resulting uncertainties are reported in the (Supplementary Figure S22; Supplementary Table S2). This allows for the determination of the exchange current density in the OER, *j*_0,OER_, for the various materials (see Table 6). *j*_0,OER_ is a crucial figure of merit to gauge the intrinsic kinetic performance of the active sites of the various samples in the OER. It should be noted that the determination of the exchange current density (*j*_0,OER_) through Tafel extrapolation inherently depends on the selection of the linear fitting region and is therefore associated with a certain degree of uncertainty. In the present work, *j*_0,OER_ values are not used for absolute quantitative comparisons, but rather as semi-qualitative descriptors to identify general trends in intrinsic activity within the investigated series. (Di Noto et al., 2021). For this reason, the discussion focuses on relative differences and consistent trends among the samples, rather than on precise numerical differences in *j*_0,OER_.

The OER electrocatalytic performance of the samples is strongly influenced by their elemental composition, especially in terms of the extent of Fe substitution by Mn or Cr. Generally, a lower overpotential (*η*_OER_) corresponds to an enhanced electrocatalytic activity since a lower voltage is necessary to achieve the same current density. The Tafel slope in the OER (*β*_OER_), which reflects the rate of electron transfer and migration during the electrocatalytic process, also influences the kinetics: lower Tafel slopes correspond to faster reaction kinetics, resulting in higher current densities at a given potential (Fabbri et al., 2014). It should be emphasised that the Tafel slope evaluated at 2 µA·cm_ECSA_^-2^ does not represent the purely kinetic regime, but is used here as a comparative descriptor under normalised conditions for all materials. In this study, the *β*_OER_ and *η*_OER_ are compared to the corresponding figures exhibited by the benchmark electrocatalyst IrO_X_ (Table 6). At a current density of 2 mA·cm_geo_^−2^, *η*_OER_ decreases as follows (Figure 8): NiFe_2_O_4_ ≈ NiFe_1.75_Cr_0.25_O_4_ > NiFe_1.75_Mn_0.25_O_4_ ≈ NiFeMnO_4__calc > IrO_x_ ≈ NiFeCrO_4_. Conversely, at the specific current density 2 μA·cm_ECSA_^-2^, *β*_OER_ decreases in the order (Table 6): NiFe_1.75_Cr_0.25_O_4_ > NiFe_2_O_4_ > NiFeCrO_4_ > IrO_x_ > NiFe_1.75_Mn_0.25_O_4_ ≈ NiFeMnO_4__calc. In the Cr-containing samples, the effect of Fe substitution follows quite a complex trend. At lower substitution levels (NiFe_1.75_Cr_0.25_O_4_), the OER performance in terms of E @ j = 2 mA·cm_geo_^−2^ is unremarkable. Instead, at higher substitution levels (NiFeCrO_4_), the OER performance improves dramatically, approaching that of the benchmark IrO_X_, on one hand thanks to the high SSA. It is highlighted that at a current density of 2 μA·cm_ECSA_^-2^ the substitution of Fe with Mn yields faster OER kinetics in comparison with IrO_X_, as witnessed by the lower *β*_OER_. To better understand the correlation between the physicochemical features of the materials and their OER performance, it is necessary to consider both the *“absolute”* OER current and the number of active sites for the OER. This is done taking into account figures of merit such as η_OER_ @ *j*_s_ = 2 μA·cm_ECSA_^-2^ and *j*_0,OER_ (Table 6).

For the Mn-doped samples (NiFe_1.75_Mn_0.25_O_4_ and NiFeMnO_4__calc), the intrinsic kinetic performance is degraded compared to the NiFe_2_O_4_ baseline. Their *j*_0,OER_ ​ values (10^−8.93^ and 10^−8.82^ mA·cm_ECSA_^-2^​, respectively) are significantly lower than that of NiFe_2_O_4_ (10^−8.31^ mA·cm_ECSA_^-2^​). This suggests that the observed improvement in apparent activity (Figure 8) is almost entirely attributable to the moderate increase in SSA (67 and 84 m^2^/g for NiFe_1.75_Mn_0.25_O_4_ and NiFeMnO_4__calc, respectively), which provides a larger number of active sites. This may be attributed to a possible lower activity of Mn cations compared to Fe, as well as to the nanoscale compositional inhomogeneity observed by EDX, which suggests the formation of segregated, less active phases. In contrast, substitution with Cr reveals a complex, non-linear effect on the kinetic properties. At a low substitution level (NiFe_1.75_Cr_0.25_O_4_), the intrinsic kinetic performance (*j*_0,OER_ = 10^−8.81^ mA·cm_ECSA_^-2^) is the lowest of the series, even lower than the Mn-doped samples. However, this poor intrinsic performance is compensated by its very high SSA (191 m^2^/g), resulting in an apparent activity similar to the pristine NiFe_2_O_4_. At high substitution levels (NiFeCrO_4_), the trend reverses. The intrinsic kinetics are significantly enhanced, with *j*_0,OER_ = 10^−7.82^ mA·cm_ECSA_^-2^, approaching the IrO_X_ benchmark (*j*_0,OER_ = 10^−7.18^ mA·cm_ECSA_^-2^). Therefore, the NiFeCrO_4_ sample is hypothesised to benefit from a powerful dual advantage: enhanced intrinsic activity combined with the highest SSA (226 m^2^/g) of the entire series. This synergy of favourable chemical composition and morphology explains its outstanding apparent performance (*η*_OER_), which approaches the IrO_X_ benchmark under the investigated conditions. It should be highlighted that the present study focuses on intrinsic electrochemical behavior and structure-property correlations under *“ex-situ”* conditions. A comprehensive durability assessment in device-relevant configurations, including long-term stability tests and *“post-operando”* characterisation, represents a subsequent step beyond the scope of this fundamental investigation.

The exceptionally high intrinsic activity of NiFeCrO_4_ is intrinsically linked to the modulation of the electronic structure induced by Cr. As observed in previous studies on Cr-substituted ferrites, the introduction of Cr^3+^significantly enhances electrocatalytic activity compared to base oxides (Singh et al., 2006; Liu et al., 2020; Liu et al., 2024; Wang et al., 2024). Raman spectroscopy suggests a specific cation distribution where the strong preference of Cr^3+^ for O_h_ sites forces a redistribution of Ni and Fe cations. As suggested by a recent publication, this particular arrangement likely facilitates the formation of an efficient electron transport (e.g., Cr(O_h_)-O-Ni(O_h_)) pathway within the spinel lattice that contribute to the high OER activity observed (Liu et al., 2024). Furthermore, the presence of Cr appears to be crucial for OER activity due to its ability to regulate the d-orbital electronic configuration. The introduction of Cr in the lattice of NiFe_2_O_4_ has been shown to shift the d-band centre closer to the Fermi energy (from −1.49 eV to −1.06 eV), thereby optimising the adsorption energy of oxygenated intermediates of the OER (*O, *OH, and *OOH) preventing the catalyst surface from binding these species too strongly or too weakly (Liu et al., 2024). Additionally, the presence of Cr enhances Metal-Oxygen covalency, which promotes the charge transfer between the transition metal cation and the oxygen adsorbent improving the kinetic of the rate-determining step of the OER (Zhai et al., 2025). Moreover, recent studies on Cr-containing spinel oxides suggest a surface reconstruction process driven by the applied anodic potential during the OER into highly catalytically active metal oxyhydroxides (e.g., NiOOH and FeOOH), a process experimentally shown to be facilitated by the presence of Cr (He et al., 2025a; Zhai et al., 2025; Wang et al., 2024; Liu et al., 2025). It was also hypothesised that Cr could undergo a leaching process, creating abundant cation and oxygen vacancies, thereby increasing the electrochemically active surface area and exposing a higher density of active sites (Duan et al., 2021; He et al., 2025b; Chen et al., 2021). Consequently, this synergistic effect of surface restructuring and electronic structure engineering, which optimises the adsorption energies of OER intermediates, results in the enhanced catalytic performance observed in the sample NiFeCrO_4_.

## 4 Conclusion

To address the critical need for sustainable, PGM-free electrocatalysts for water splitting, this study presents a series of Cr- and Mn-doped NiFe_2_O_4_ spinel oxides via a subcritical hydrothermal method. The presented low-temperature synthetic approach allows to overcome the intrinsic limitation of common solid-state routes, leading to the obtainment of materials in the form of nanoparticles, characterised by reduced crystallite sizes and with a very high specific surface area. A comprehensive structural and physicochemical characterisation elucidated the distinct impacts of the two dopants on the host lattice. Cr was successfully incorporated into the spinel lattice, inducing a significant reduction in particle size (down to <5 nm for NiFeCrO_4_) and a consequent maximisation of the specific surface area (226 m^2^/g). Conversely, doping with Mn presented synthetic challenges. While low concentrations were accommodated into the spinel lattice, high degrees of Fe substitution with Mn led to the segregation of a secondary Ni(OH)_2_ phase. Although a subsequent calcination step was attempted to promote the diffusion of Ni in the spinel structure for the NiFeMnO_4_ sample, EDX analyses suggest a persistent nanoscale inhomogeneity and surface Ni enrichment, highlighting the difficulties in introducing a high Mn content within the spinel structure via this specific hydrothermal route.

Electrochemical investigations demonstrated that the nature of the dopant strongly influences the OER performance. Although Mn-doped samples exhibited a higher apparent activity than the baseline NiFe_2_O_4_ (as gauged by *η*_OER_ at 2 mA·cm_geo_^−2^, Figure 8; Table 6), this improvement could be attributable to an increased number of active sites (higher SSA). Indeed, with respect to baseline NiFe_2_O_4_, the intrinsic kinetics of the OER active sites for Mn-doped samples (as gauged by *j*_0,OER_ values) is lower. In contrast, the NiFeCrO_4_ sample exhibited an apparent activity (*η*_OER_ at 2 mA·cm_geo_^−2^) closely matching the benchmark IrO_X_ (Figure 8). Specifically, *η*_OER_ at 2 mA·cm_geo_^−2^ of NiFeCrO_4_ and IrO_X_ is equal to 349 and 351 mV, respectively (Table 6). This outstanding result is ascribed to a synergistic effect: the NiFeCrO_4_ sample combines the highest specific surface area of the series (226 m^2^/g) with improved intrinsic kinetics (*j*_0,OER_ = 10^−7.82^ mA·cm_ECSA_^-2^). This kinetic boost derives from an optimal surface chemistry, where the presence of Cr^3+^ (which is predicted to modulate the overall, e_g_ orbital occupation to a favourable mean value of unity) is expected to tune the electronic structure to favour the OER. Furthermore, Cr^6+^ species are prone to dissolve in the KOH solution as chromate ions (CrO_4_^2-^) generating surface vacancies and expose the underlying, catalytically active sites contributing to the high catalytic activity.

Ultimately, this study identifies NiFeCrO_4_ as a promising candidate as electrocatalyst for the OER in alkaline water electrolysis and validates electronic modulation of NiFe_2_O_4_ via Cr-doping as an effective strategy for catalyst design.

## Data Availability

The original contributions presented in the study are included in the article/Supplementary Material, further inquiries can be directed to the corresponding author.
