# Supplementary material for: Boosting the OER performance of NiFe2O4 through Cr and Mn doping via hydrothermal synthesis
Source: Front Chem. 2026 Apr 21;14:1778233. doi: 10.3389/fchem.2026.1778233 (PMC13139091; doi:10.3389/fchem.2026.1778233)
Supplement: Supplementary file 1 [file DataSheet1.docx]

Boosting the OER performance of NiFe_2_O_4_ through Cr and Mn doping via hydrothermal synthesis

Davide Vendrame^1^, Soufiane Boudjelida^2^, Enrico Negro^2^, Paolo Dolcet^1,4^, Vito Di Noto^2^, Silvia Gross^1,3,4*^

^1^ Università degli Studi di Padova, Dipartimento di Scienze Chimiche, Padova, Italy

^2^ Università degli Studi di Padova, Dipartimento di Ingegneria Industriale, Padova, Italy

^3^ INSTM, Consorzio Interuniversitario per la Scienza e Tecnologia dei Materiali, Firenze, Italy

^4^ Karlsruher Institut für Technologie, Institut für Technische Chemie und Polymerchemie, Karlsruhe, Germany

*** Correspondence:** Silvia Gross

silvia.gross@unipd.it

Supplementary Material

**Detailed Electrochemical Methods**

Measurements were conducted using a graphite rod counter electrode and a Hg/HgO/KOH (aq) (0.1 M) reference electrode. Calibration to the RHE scale was carried out by H_2_ oxidation/reduction measurements on a platinum electrode before each experiment.

For the preparation of the working electrode, 10 mg of each OER electrocatalyst were mixed with 2 mg of Vulcan™ XC72R carbon black. The latter was added to the electrocatalyst layer primarily to ensure an adequate electronic conductivity. Indeed, the addition of a conductive carbon additive minimizes possible ohmic drops across the electrocatalyst layer and improves the electrical percolation between the glassy carbon substrate and the active material particles.(Lorandi et al. 2023) This strategy allows the measured electrochemical response to better reflect the intrinsic electrocatalytic properties of the spinel oxides rather than the limitations arising from poor electronic transport within the electrocatalytic film. The resulting powders were suspended by ultrasonication in a solution consisting of 1200 μL of isopropanol, 70 μL of 0.01 M KOH (Sigma-Aldrich) and 100 μL of 5 wt% Nafion dispersion (Sigma-Aldrich). A suitable amount of the suspension was drop-cast onto the surface of the glassy carbon disk (area = 0.237 cm^2^) of a rotating disk electrode (RDE), achieving a loading of 0.185 mg·cm^-2^ and acting as the working electrode. Prior to the deposition, the glassy carbon (GC) disk was mechanically polished using an alumina slurry (0.05 μm), thoroughly rinsed with deionized water and finally dried under ambient conditions. After drop-casting the suspension, the electrode was dried in air while spinning the RDE tip at 700 rpm until complete solvent evaporation.(Lorandi et al. 2023)

All potentials are reported versus the reversible hydrogen electrode (RHE) scale. Calibration was carried out by H_2_ oxidation/reduction measurements on a platinum electrode before each experiment. The overpotentials for the OER, 𝜂_OER_, were calculated according to Equation (1):

1. 𝜂_OER_ = E_RHE_ – E_0_

E_0_ is the thermodynamic potential of the OER. The polarization curves for OER were recorded in Linear Sweep Voltammetry (LSV) mode at a scan rate of 5 mV/s. The LSV curves reported in the manuscript correspond to the positive-going (anodic) scans. Prior to LSV measurements, the electrodes were conditioned by cyclic voltammetry in the potential range 1.2–2.2 V *vs.* RHE at a sweep rate of 100 mV/s. Cycling was stopped when the OER profiles reached stability. For each sample, reproducibility was assessed by performing three LSV measurements on the same conditioned working electrode, which resulted in highly consistent polarization curves. Experimentally-measured potentials were corrected for the ohmic drop corresponding to the uncompensated solution resistance (Ru), determined from impedance measurements.(van der Vliet et al. 2010)

The extrapolation of the linear fitting used to determine the exchange current density *j*_0,OER_ was carried out for each of the curves shown in Figure 10 and Supplementary Figure S22. The fitting was performed over a window with a width of approximately 0.5 μA·$\mathrm{cm}_{\mathrm{ECSA}}^{-2}$ starting from the lowest current density displayed in Figure 10 for each curve. The details of the linear fitting are reported in the Supplementary Table S2. The uncertainty on *j*_0,OER_ was estimated by standard error propagation from the uncertainties of the slope and intercept of the linear Tafel fit, neglecting covariance terms. The resulting uncertainties on log_10_(*j*_0,OER_) are significantly smaller than the differences observed among the electrocatalysts and therefore do not affect the trends discussed in the manuscript.





**Figure S1.** X-ray diffractogram of the sample NiFeMnO_4_ synthetized by the hydrothermal method at 250 °C. The blue lines correspond to the spinel oxide structure (Fe_3_O_4_ pattern) and the red lines to the secondary phase formed by carbonates (MnCO_3_ pattern).

Table S1. Parameters used for the calculation of the mean crystallite diameter using the Scherrer’s equation.

| Composition | Obs. Max (°) | FWHM (°) | k | Instr. Width (°) | Crystallite (nm) |
| --- | --- | --- | --- | --- | --- |
| NiFe_2_O_4_ | 35.64 | 0.285 | 0.89 | 0.05 | 29 |
| NiFe_1.75_Cr_0.25_O_4_ | 35.69 | 0.193 | 0.89 | 0.05 | 44 |
| NiFe_1.5_Cr_0.5_O_4_ | 35.67 | 0.137 | 0.89 | 0.05 | 65 |
| NiFeCrO_4_ | 35.73 | 2.822 | 0.89 | 0.05 | 3 |
| NiFe_1.75_Mn_0.25_O_4_ | 35.58 | 0.415 | 0.89 | 0.05 | 20 |
| NiFe_1.5_Mn_0.5_O_4_ | 35.51 | 0.425 | 0.89 | 0.05 | 20 |
| NiFeMnO_4_ | 35.39 | 0.707 | 0.89 | 0.05 | 12 |
| NiFeMnO_4__calc | 35.41 | 0.507 | 0.89 | 0.05 | 16 |


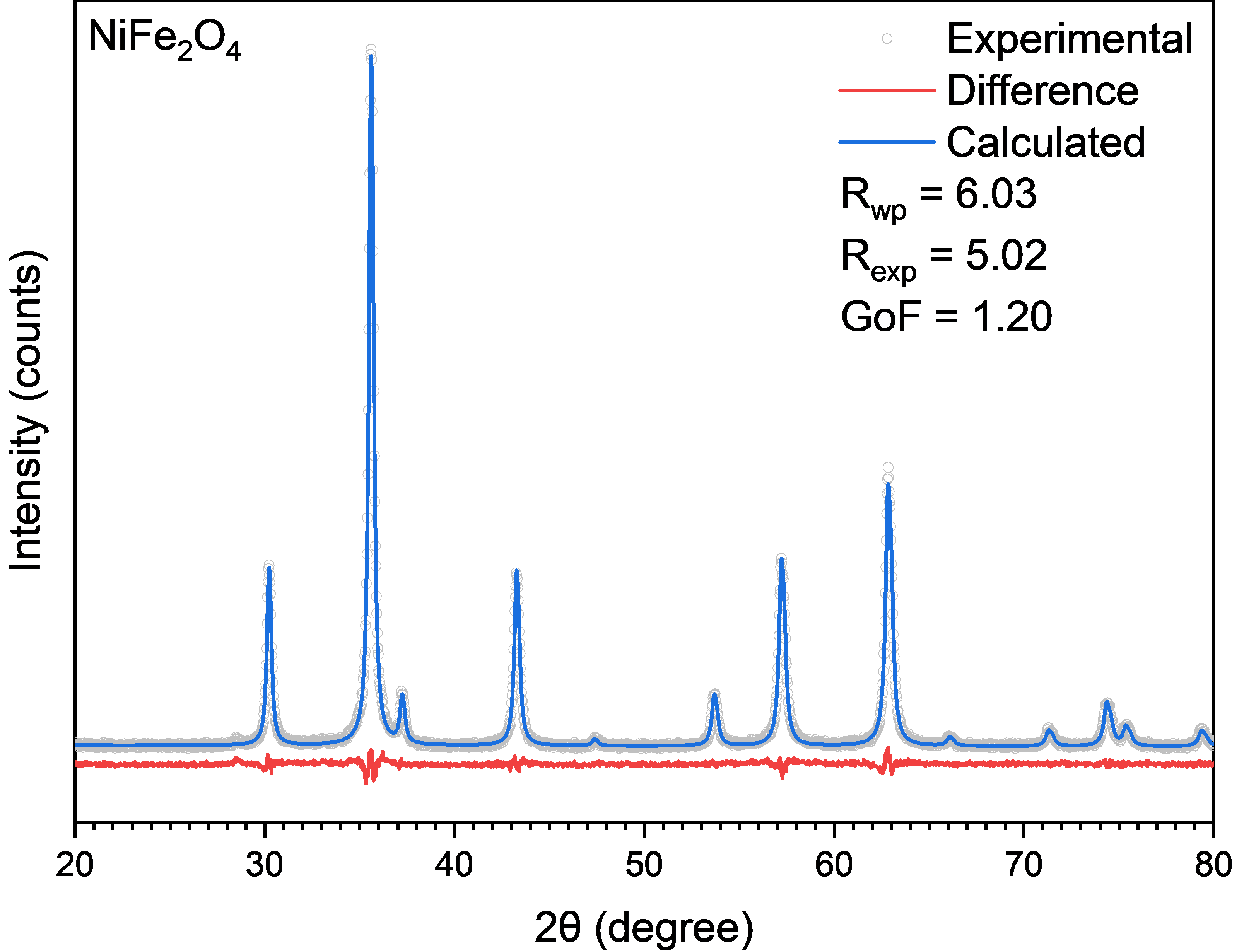


Supplementary Figure S2. Pawley refinement of the XRD data for the NiFe_2_O_4_ sample. Experimental data are shown as grey circles, the blue line represents the calculated pattern, and the red line shows the difference between the experimental and calculated profiles.


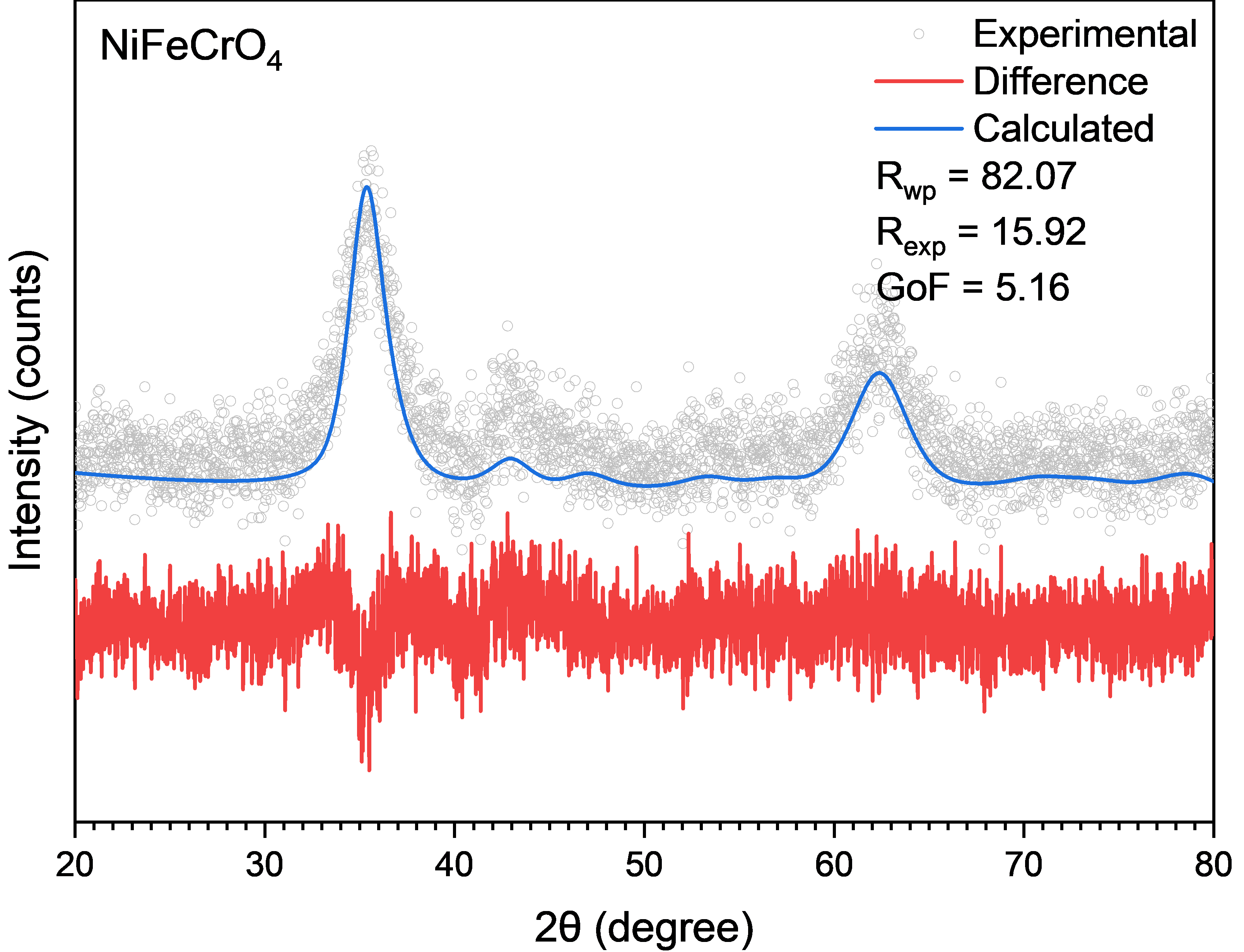


Supplementary Figure S3. Pawley refinement of the XRD data for the NiFeCrO_4_ sample. Experimental data are shown as grey circles, the blue line represents the calculated pattern, and the red line shows the difference between the experimental and calculated profiles.


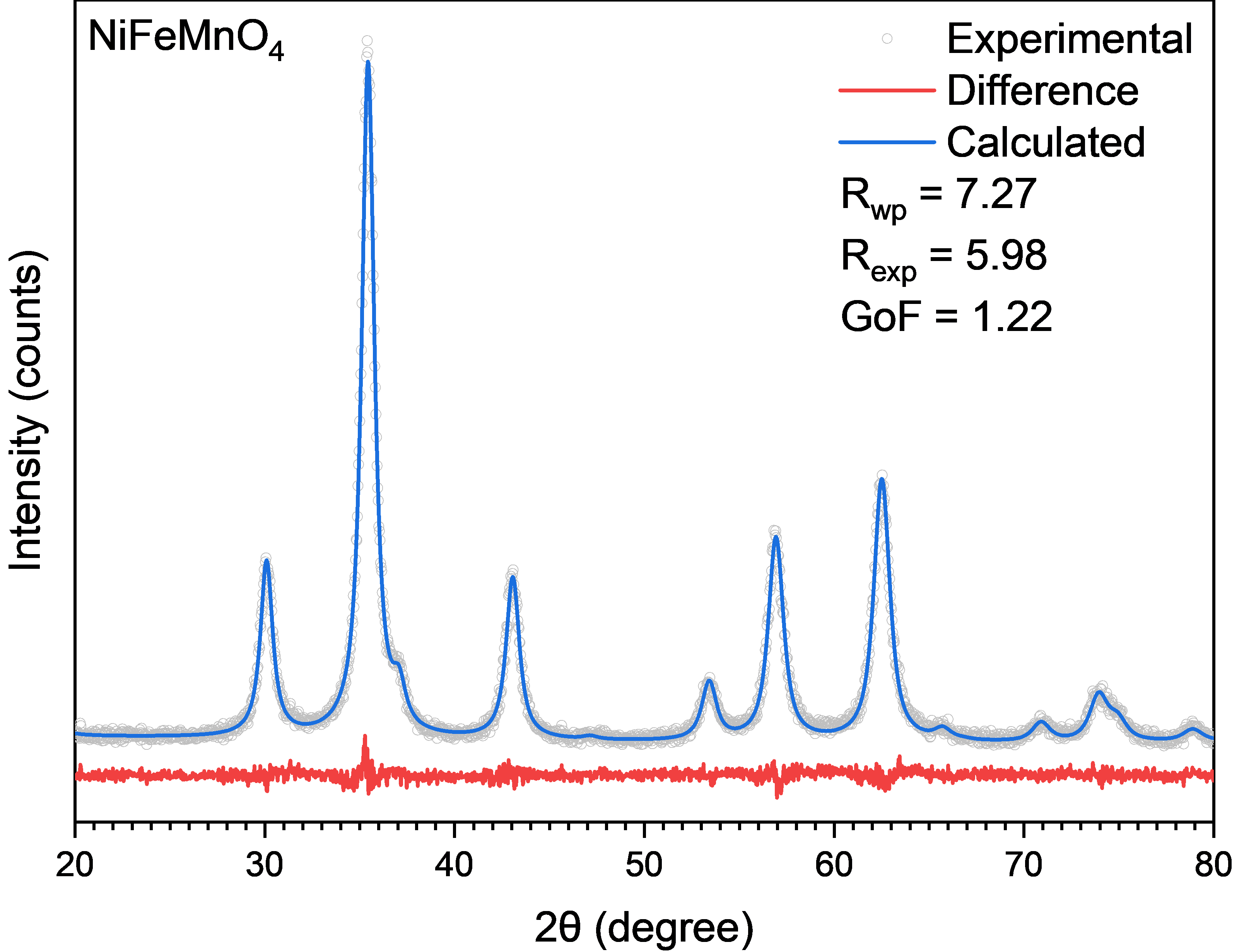


Supplementary Figure S4. Pawley refinement of the XRD data for the NiFeMnO_4__calc sample. Experimental data are shown as grey circles, the blue line represents the calculated pattern, and the red line shows the difference between the experimental and calculated profiles.


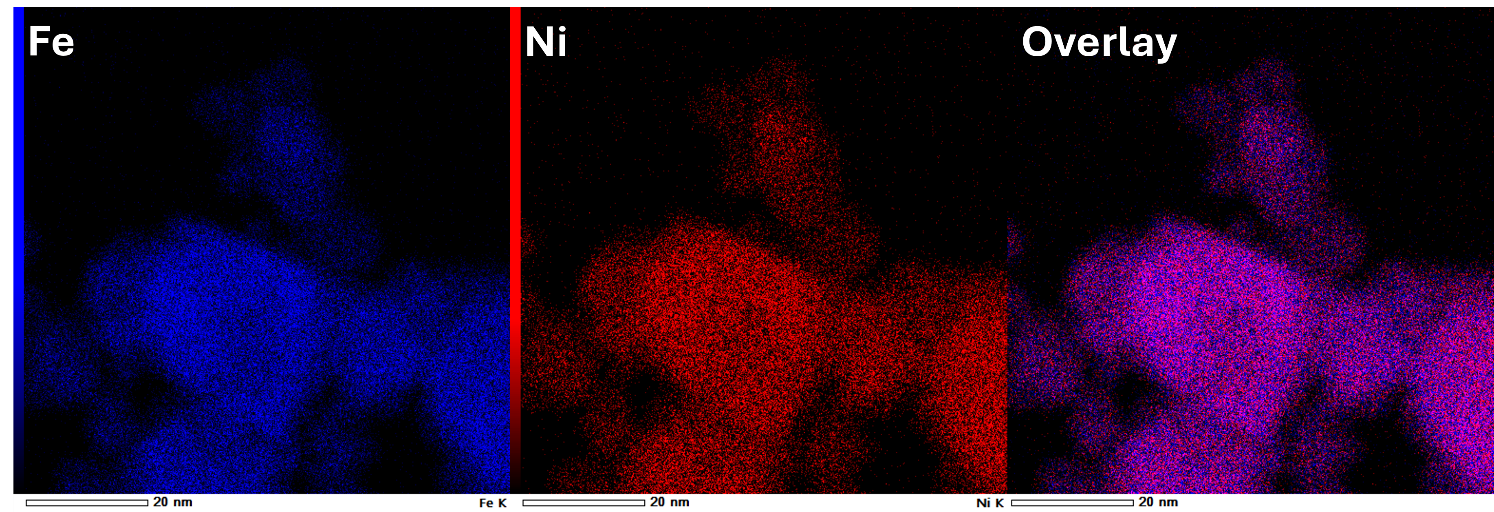


Supplementary Figure S5. EDX elemental mapping showing the spatial distribution of Fe (blue) and Ni (red). The overlapped image highlights the co-localization and distribution of these elements across the NiFe_2_O_4_ sample.


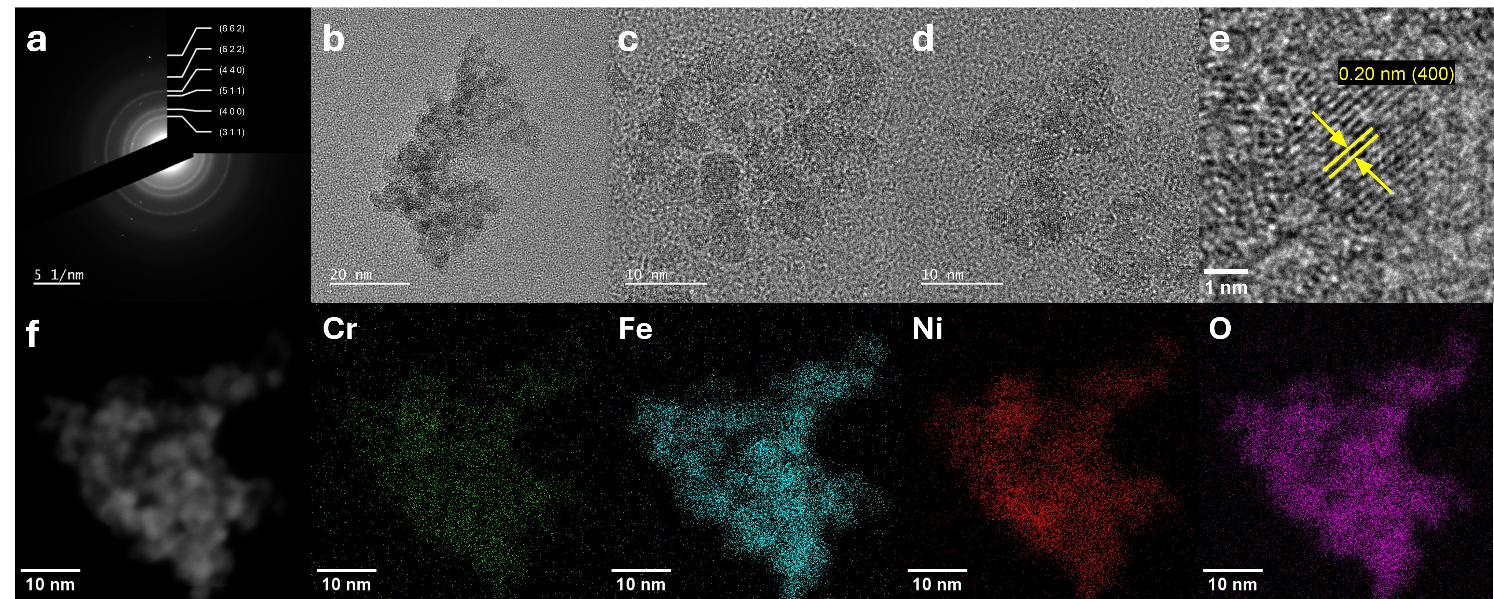


Supplementary Figure S6. TEM, SAED, and EDX results of the sample NiFe_1.75_Cr_0.25_O_4_: (a) SAED pattern, (b, c, d, e) TEM micrographs, and (f) EDX elemental mapping.


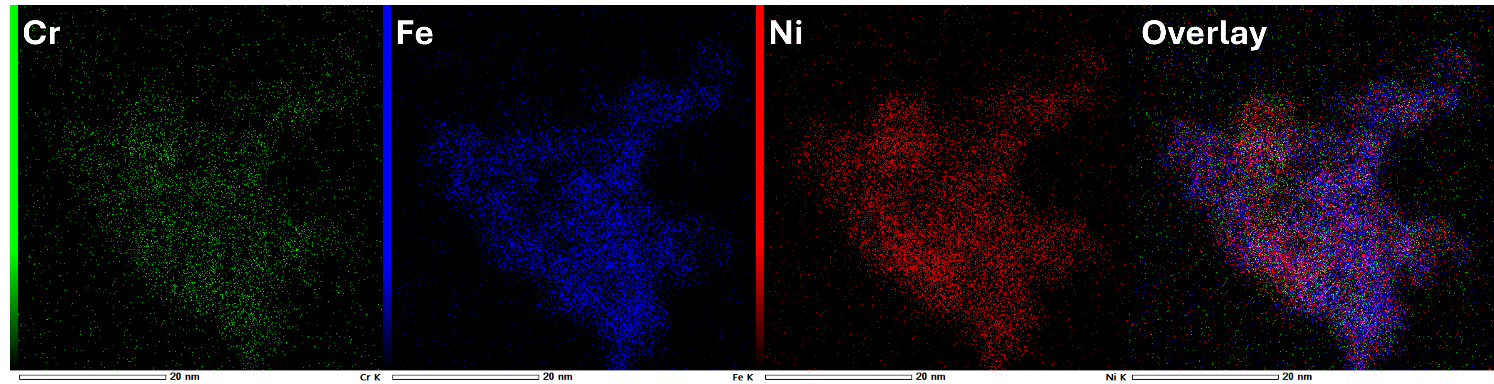


Supplementary Figure S7. EDX elemental mapping showing the spatial distribution of Cr (green), Fe (blue), and Ni (red). The overlapped image highlights the co-localization and distribution of these elements across the NiFe_1.75_Cr_0.25_O_4_ sample.


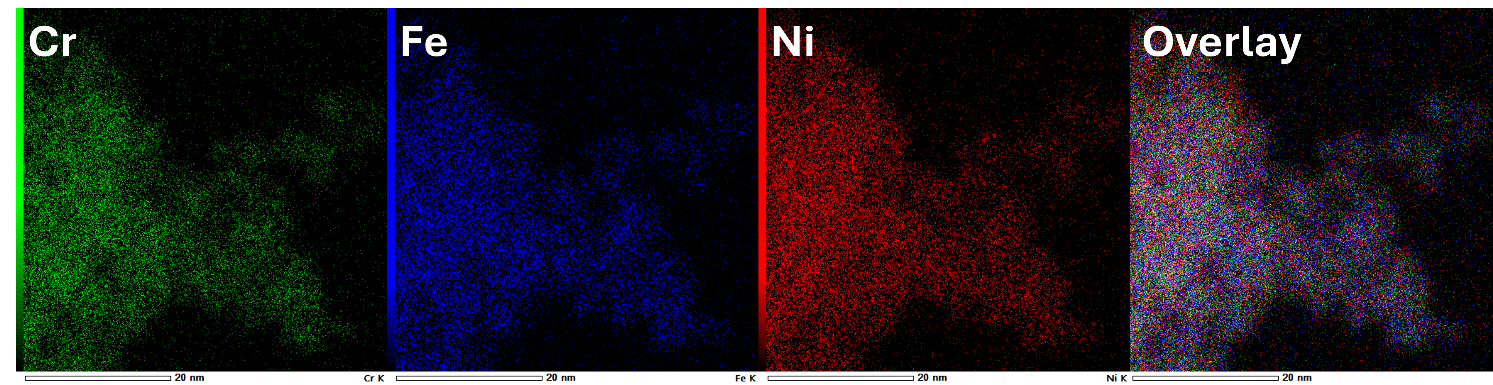


Supplementary Figure S8. EDX elemental mapping showing the spatial distribution of Cr (green), Fe (blue), and Ni (red). The overlapped image highlights the co-localization and distribution of these elements across the NiFeCrO_4_ sample.


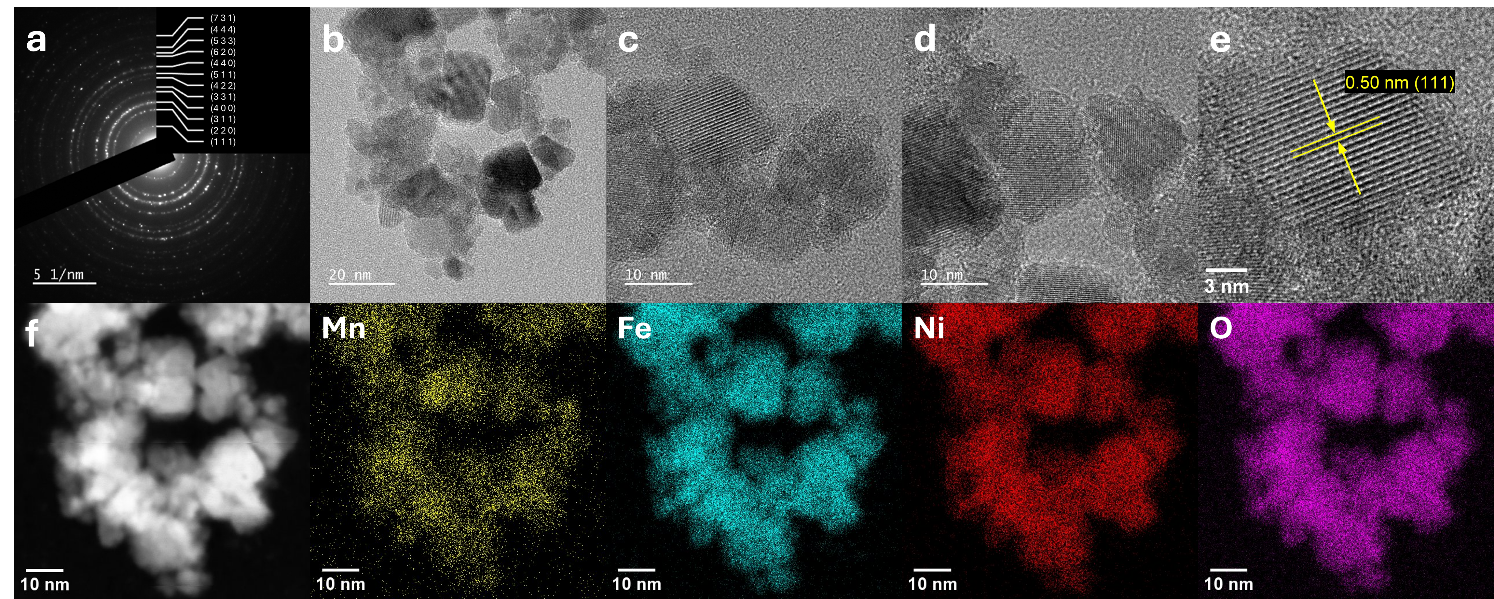


Supplementary Figure S9. TEM, SAED, and EDX results of the sample NiFe_1.75_Mn_0.25_O_4_: (a) SAED pattern, (b, c, d, e) TEM micrographs, and (f) EDX elemental mapping.


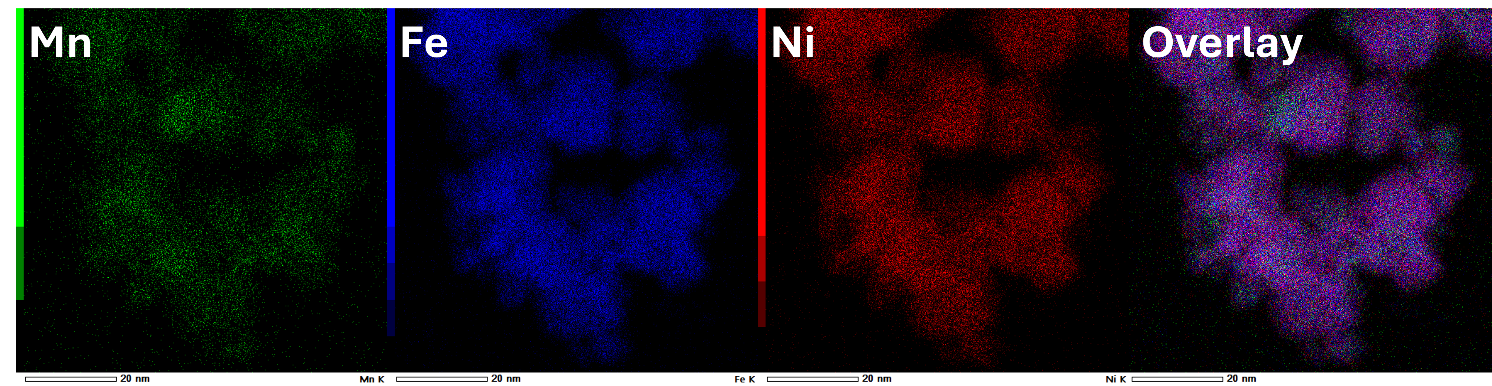


Supplementary Figure S10. EDX elemental mapping showing the spatial distribution of Mn (green), Fe (blue), and Ni (red). The overlapped image highlights the co-localization and distribution of these elements across the NiFe_1.75_Mn_0.25_O_4_ sample.


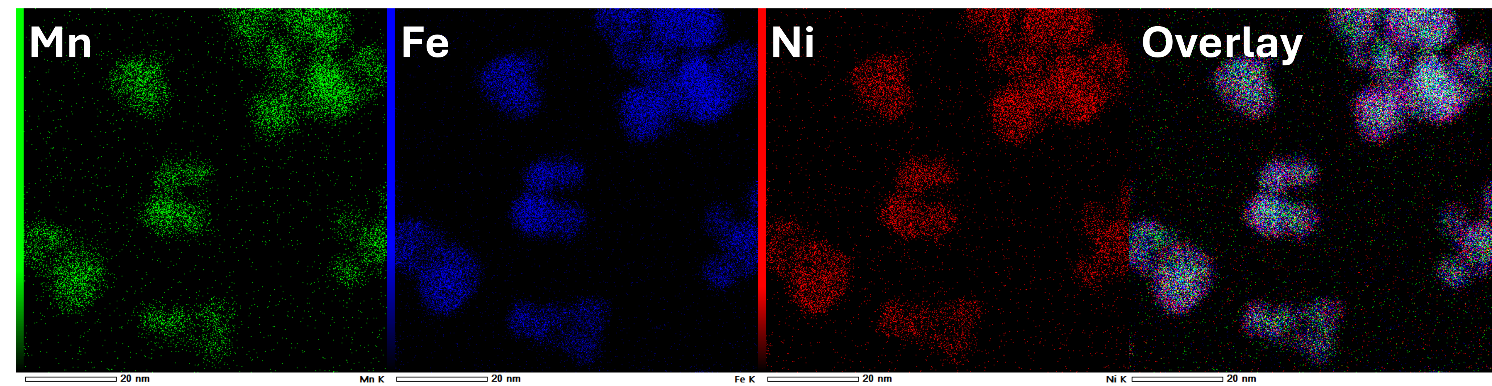


Supplementary Figure S11. EDX elemental mapping showing the spatial distribution of Mn (green), Fe (blue), and Ni (red). The overlapped image highlights the co-localization and distribution of these elements across the NiFeMnO_4__calc sample.


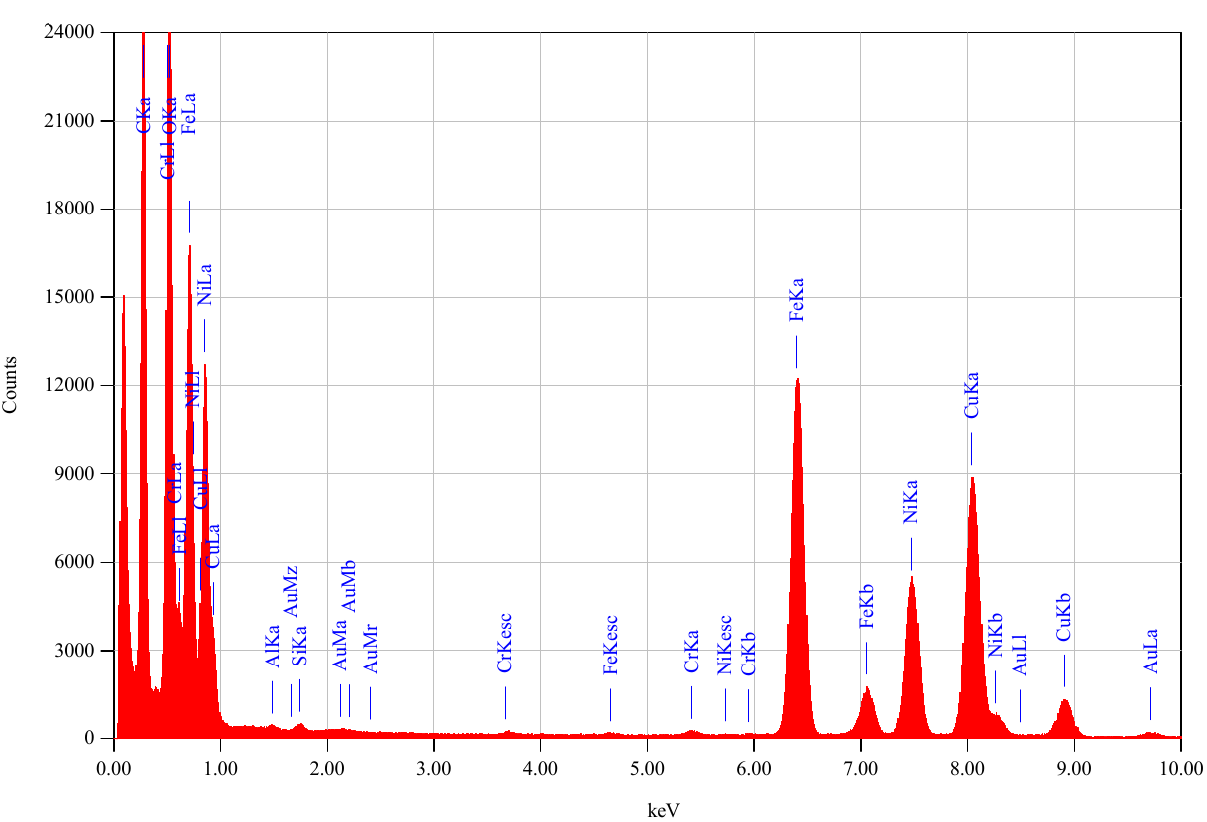


Supplementary Figure S12. EDX spectrum for the sample NiFe_2_O_4_.


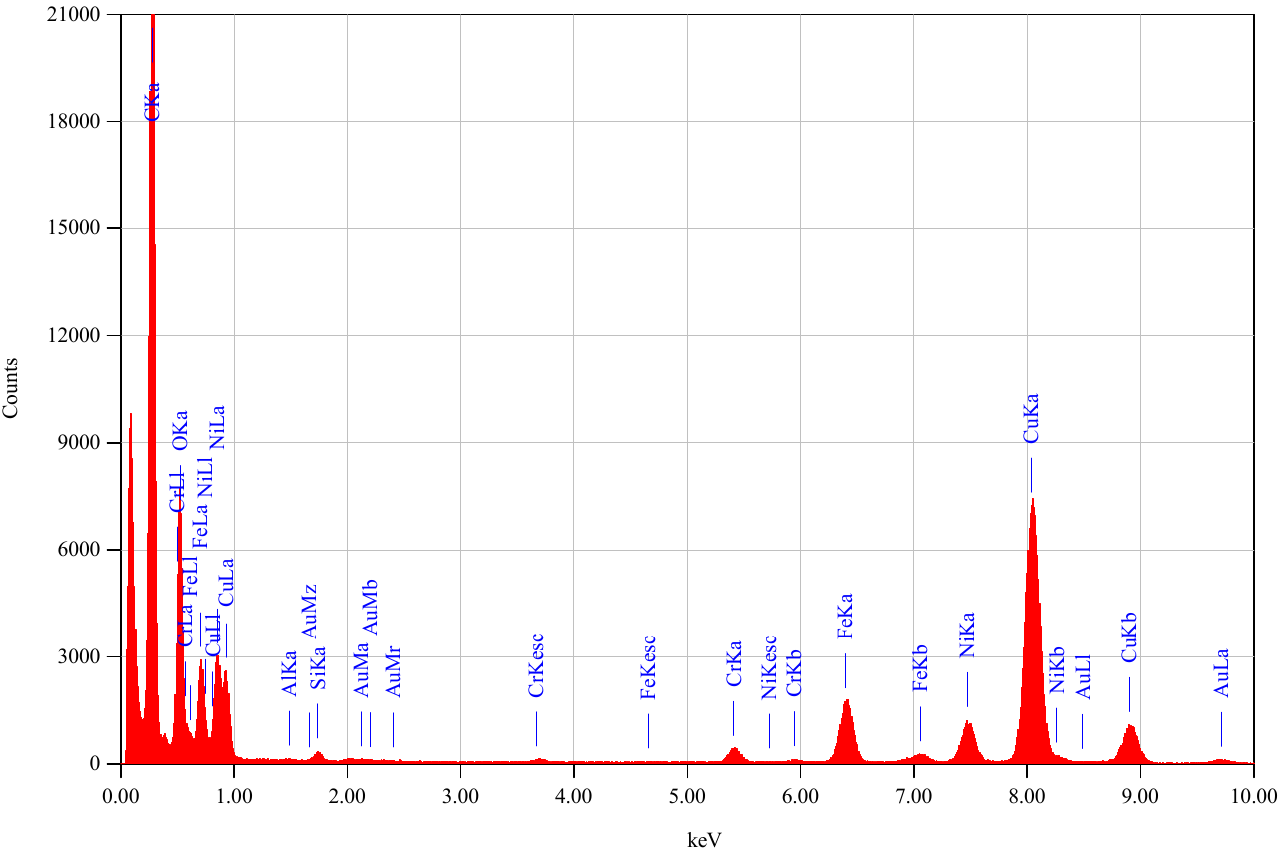


Supplementary Figure S13. EDX spectrum for the sample NiFe_1.75_Cr_0.25_O_4_.


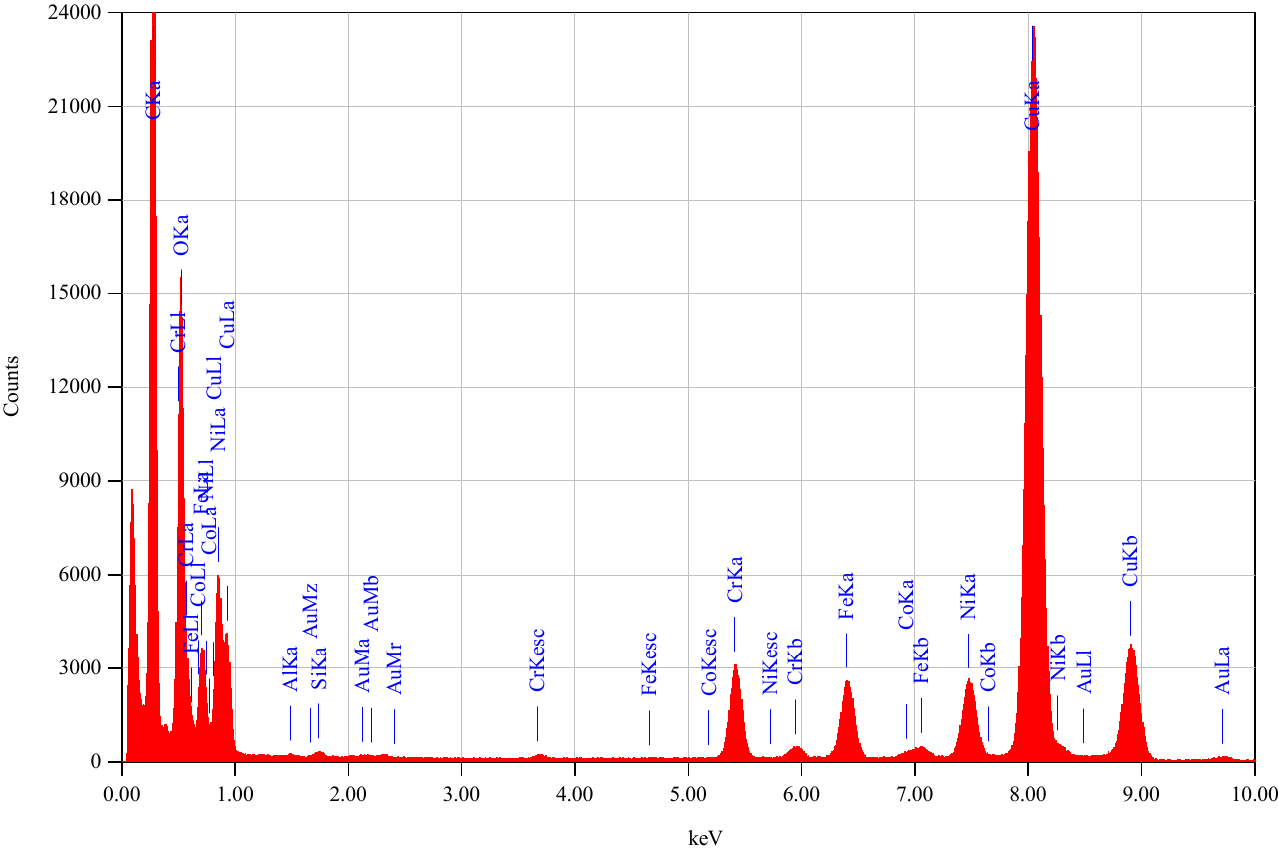


Supplementary Figure S14. EDX spectrum for the sample NiFeCrO_4_.


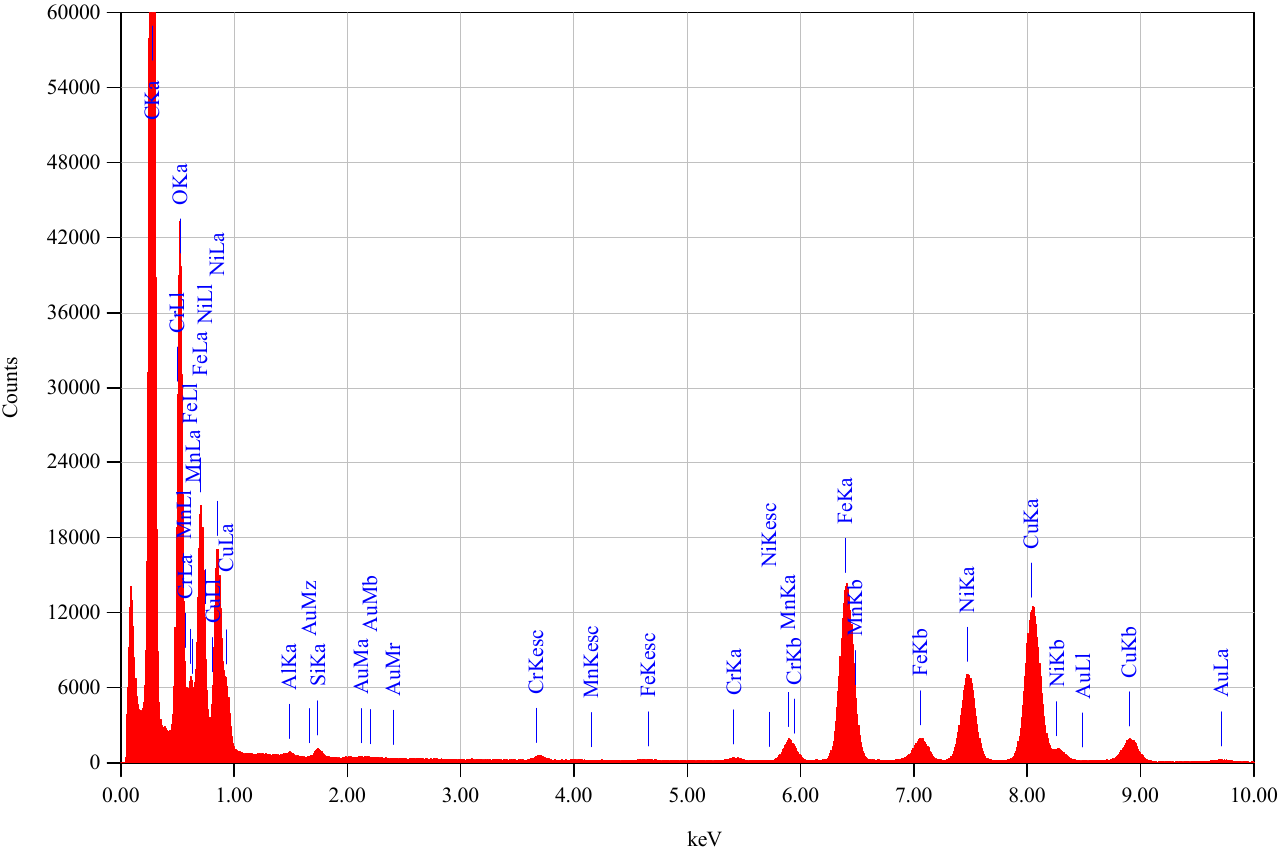


Supplementary Figure S15. EDX spectrum for the sample NiFe_1.75_Mn_0.25_O_4_.


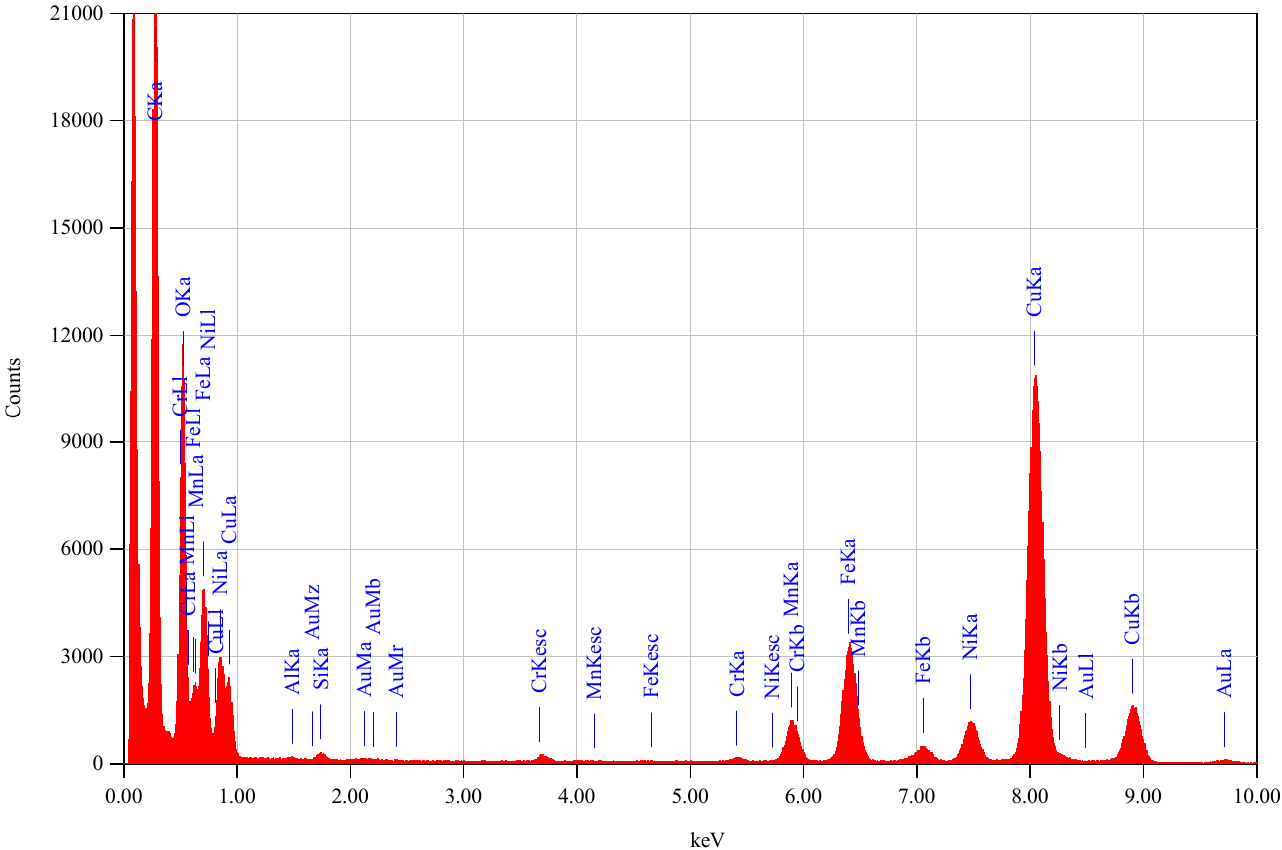


Supplementary Figure S16. EDX spectrum for the sample NiFeMnO_4__calc.


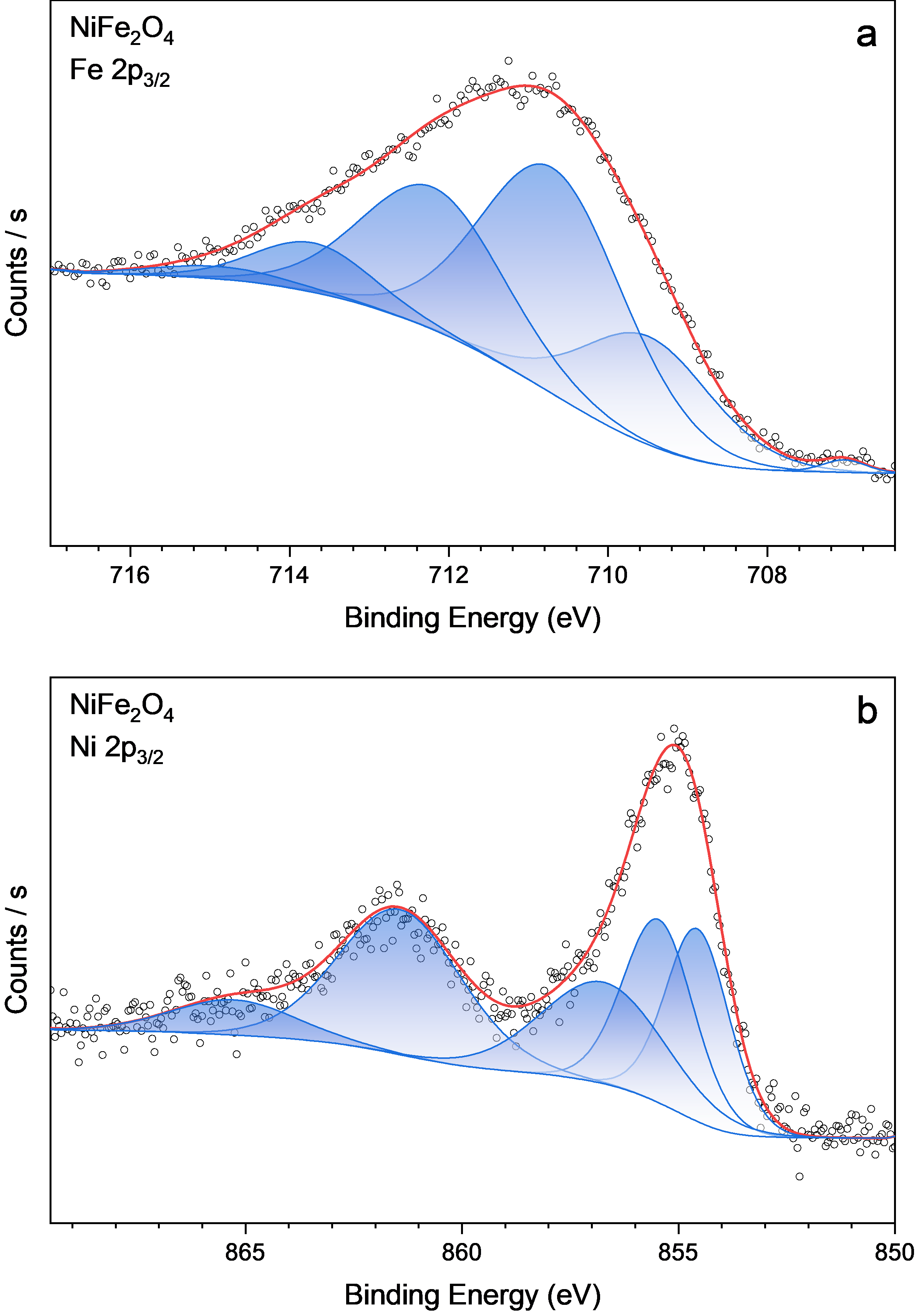


Supplementary Figure S17. XPS spectra fitting of the sample NiFe_2_O_4_ in the a) Fe 2p_3/2_ region and b) Ni 2p_3/2_ region. The spectra were fitted using the multiplet structure proposed by (Biesinger et al. 2011).


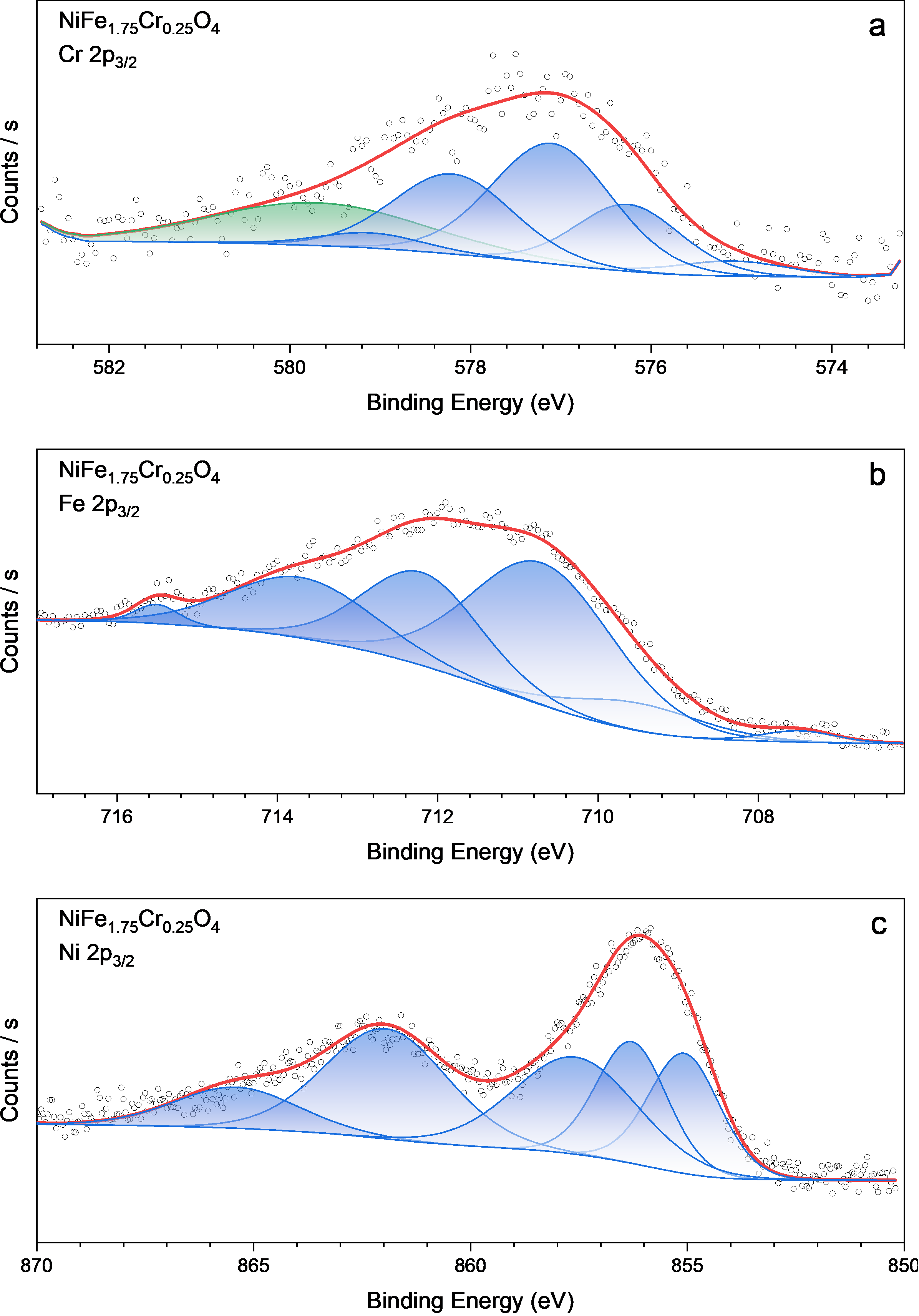


Supplementary Figure S18. XPS spectra fitting of the sample NiFe_1.75_Cr_0.25_O_4_ in the a) Cr 2p region, b) Fe 2p region, and c) Ni 2p region. The Cr 2p spectrum confirms the presence of Cr^3+^ (blue curves) and Cr^6+^ (green curves) species.(Justin Gorham 2012) The Fe 2p and Ni 2p spectra were fitted using the multiplet structure proposed by (Biesinger et al. 2011).


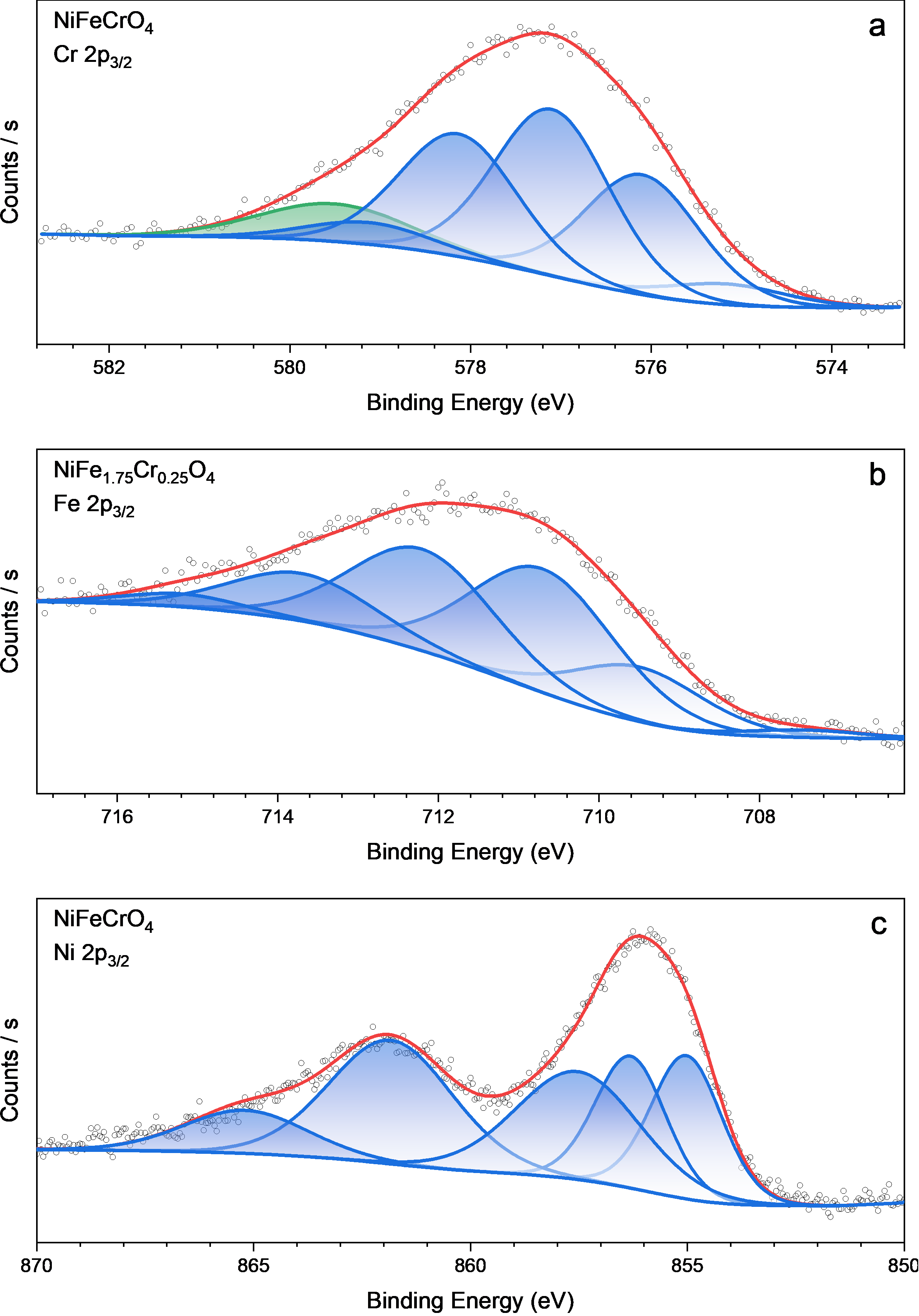


Supplementary Figure S19. XPS spectra fitting of the sample NiFeCrO_4_ in the a) Cr 2p region, b) Fe 2p region, and c) Ni 2p region. The Cr 2p spectrum confirms the presence of Cr^3+^ (blue curves) and Cr^6+^ (green curves) species.(Justin Gorham 2012) The Fe 2p and Ni 2p spectra were fitted using the multiplet structure proposed by (Biesinger et al. 2011).


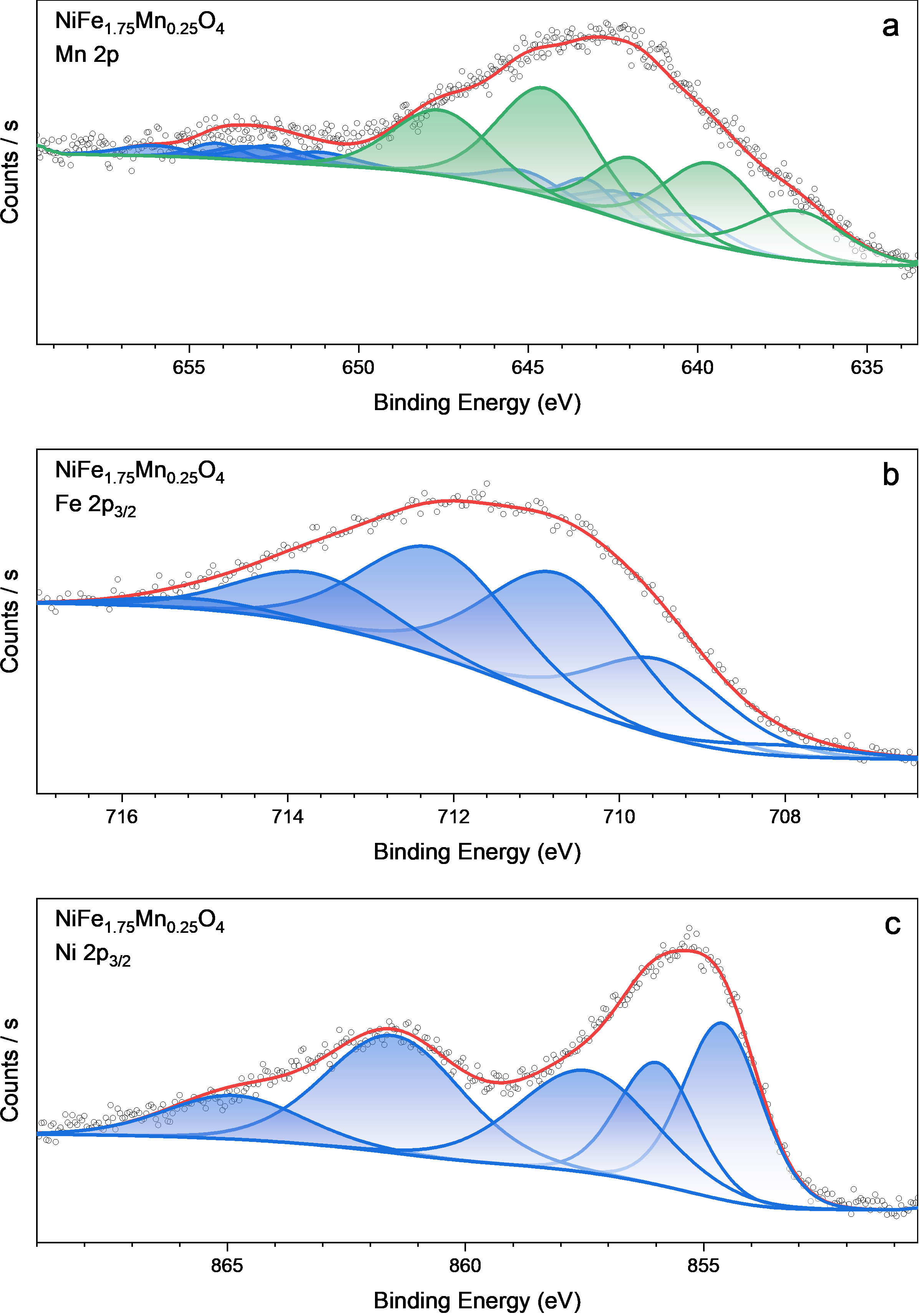


Supplementary Figure S20. XPS spectra fitting of the sample NiFe_1.75_Mn_0.25_O_4_ in the a) Mn 2p region, b) Fe 2p region, and c) Ni 2p region. The Mn 2p spectrum was fitted taking into account the Ni Auger signals (green curves) and the multiplet structure (blue lines) proposed by (Biesinger et al. 2011). The Fe 2p and Ni 2p spectra were fitted using the multiplet structure proposed by (Biesinger et al. 2011).


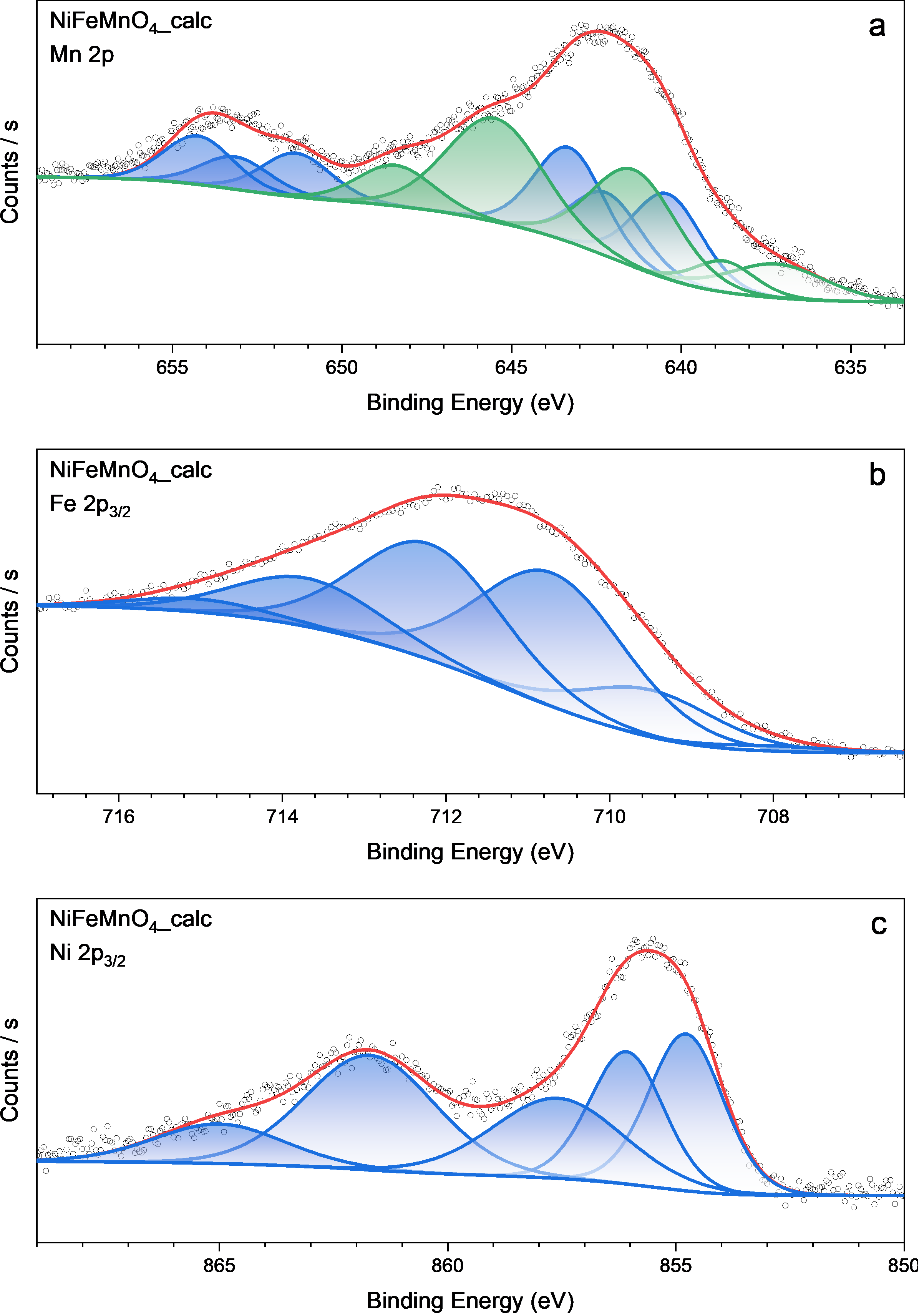


Supplementary Figure S21. XPS spectra fitting of the sample NiFeMnO_4__calc in the a) Mn 2p region, b) Fe 2p region, and c) Ni 2p region. The Mn 2p spectrum was fitted taking into account the Ni Auger signals (green curves) and the multiplet structure (blue lines) proposed by (Biesinger et al. 2011). The Fe 2p and Ni 2p spectra were fitted using the multiplet structure proposed by (Biesinger et al. 2011).


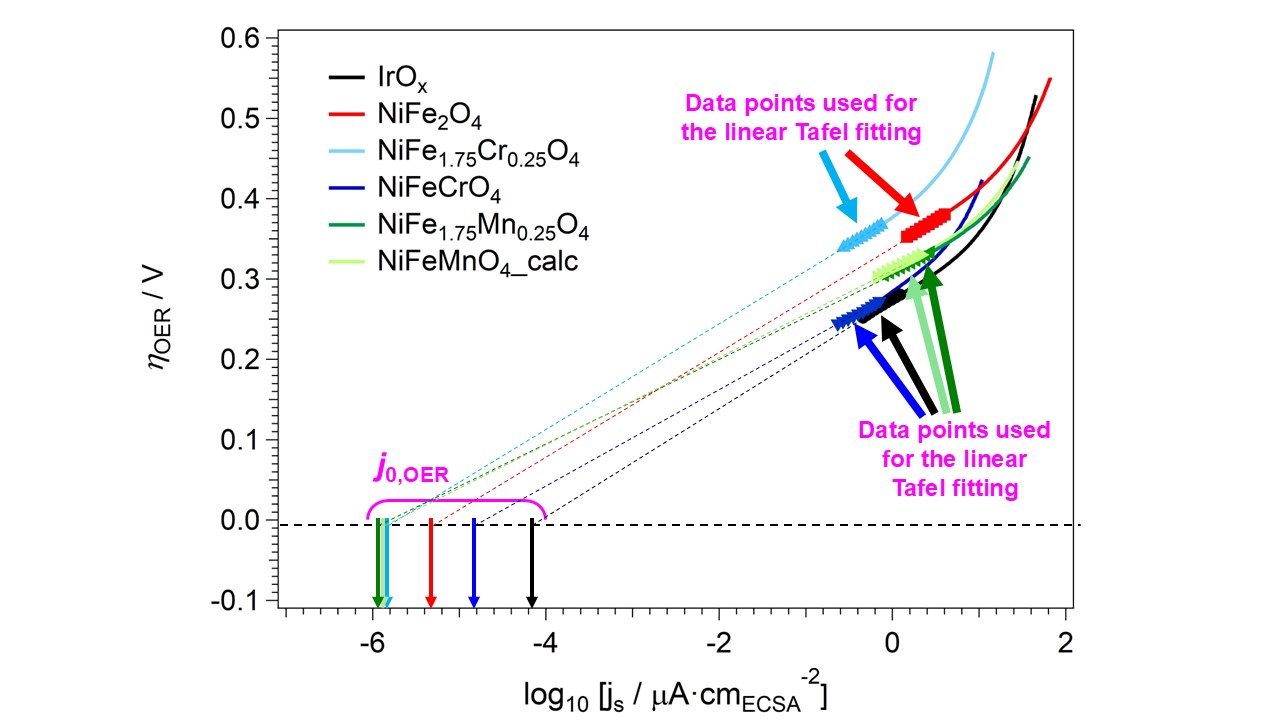


Supplementary Figure S22. Determination of *j*_0,OER_ for the proposed materials by extrapolation of the Tafel plots to *η*_OER_ = 0. The linear fitting regions used for the determination of *j*_0,OER_ are indicated.

Supplementary Table S2. Linear fitting parameters used for the determination of *j*_0,OER_ and propagation of the associated uncertainties.

| **Sample** | **Slope (V·dec^-1^)** | **Intercept (V)** | **R^2^** | ***j*_0,OER_ (mA·**$\mathbf{cm}_{\mathbf{ECSA}}^{\mathbf{-2}}$**)** |
| --- | --- | --- | --- | --- |
| **NiFe_2_O_4_** | 0.064348 ± 0.000268 | 0.34176 ± 0.000113 | 0.9997 | 10^-(8.31±0.02)^ |
| **NiFe_1.75_Cr_0.25_O_4_** | 0.064616 ± 0.000401 | 0.37517 ± 0.000143 | 0.9993 | 10^-(8.81±0.04)^ |
| **NiFeCrO_4_** | 0.058158 ± 0.000835 | 0.28037 ± 0.00033 | 0.9963 | 10^-(7.82±0.07)^ |
| **NiFe_1.75_Mn_0.25_O_4_** | 0.052214 ± 0.000254 | 0.30971 ± 0.0000661 | 0.9996 | 10^-(8.93±0.03)^ |
| **NiFeMnO_4__calc** | 0.053732 ± 0.000213 | 0.31236 ± 0.0000398 | 0.9997 | 10^-(8.82±0.02)^ |
| **IrO_x_** | 0.065843 ± 0.000414 | 0.27512 ± 0.0000756 | 0.9993 | 10^-(7.18±0.03)^ |

**Bibliography**

Biesinger, Mark C., Brad P. Payne, Andrew P. Grosvenor, Leo W. M. Lau, Andrea R. Gerson, and Roger St. C. Smart. 2011. “Resolving Surface Chemical States in XPS Analysis of First Row Transition Metals, Oxides and Hydroxides: Cr, Mn, Fe, Co and Ni.” *Applied Surface Science* 257 (7): 2717–30. https://doi.org/10.1016/j.apsusc.2010.10.051.

Justin Gorham. 2012. “NIST X-Ray Photoelectron Spectroscopy Database - SRD 20.” Version 1.0.4. With Justin Gorham. National Institute of Standards and Technology, October 10. https://doi.org/10.18434/T4T88K.

Lorandi, Francesca, Keti Vezzù, Angeloclaudio Nale, et al. 2023. “Tuning Synthesis Parameters and Support Composition for High-Performing and Durable *Core-Shell* Pt–Ni Carbon Nitride Electrocatalysts for the Oxygen Reduction Reaction.” *Journal of Power Sources* 555 (January): 232390. https://doi.org/10.1016/j.jpowsour.2022.232390.

Vliet, Dennis van der, Dusan S. Strmcnik, Chao Wang, Vojislav R. Stamenkovic, Nenad M. Markovic, and Marc T. M. Koper. 2010. “On the Importance of Correcting for the Uncompensated Ohmic Resistance in Model Experiments of the Oxygen Reduction Reaction.” *Journal of Electroanalytical Chemistry* 647 (1): 29–34. https://doi.org/10.1016/j.jelechem.2010.05.016.
